# Signaling pathways and intervention for therapy of type 2 diabetes mellitus

**DOI:** 10.1002/mco2.283

**Published:** 2023-06-07

**Authors:** Rong Cao, Huimin Tian, Yu Zhang, Geng Liu, Haixia Xu, Guocheng Rao, Yan Tian, Xianghui Fu

**Affiliations:** ^1^ Department of Endocrinology and Metabolism State Key Laboratory of Biotherapy and Cancer Center West China Hospital Sichuan University and Collaborative Innovation Center of Biotherapy Chengdu Sichuan China; ^2^ Department of Endocrinology and Metabolism State Key Laboratory of Biotherapy and Cancer Center West China Medical School, West China Hospital Sichuan University Chengdu Sichuan China

**Keywords:** β cell dysfunction, antidiabetic drug, diabetes complications, insulin resistance, pathology, therapeutic target

## Abstract

Type 2 diabetes mellitus (T2DM) represents one of the fastest growing epidemic metabolic disorders worldwide and is a strong contributor for a broad range of comorbidities, including vascular, visual, neurological, kidney, and liver diseases. Moreover, recent data suggest a mutual interplay between T2DM and Corona Virus Disease 2019 (COVID‐19). T2DM is characterized by insulin resistance (IR) and pancreatic β cell dysfunction. Pioneering discoveries throughout the past few decades have established notable links between signaling pathways and T2DM pathogenesis and therapy. Importantly, a number of signaling pathways substantially control the advancement of core pathological changes in T2DM, including IR and β cell dysfunction, as well as additional pathogenic disturbances. Accordingly, an improved understanding of these signaling pathways sheds light on tractable targets and strategies for developing and repurposing critical therapies to treat T2DM and its complications. In this review, we provide a brief overview of the history of T2DM and signaling pathways, and offer a systematic update on the role and mechanism of key signaling pathways underlying the onset, development, and progression of T2DM. In this content, we also summarize current therapeutic drugs/agents associated with signaling pathways for the treatment of T2DM and its complications, and discuss some implications and directions to the future of this field.

## Graphical abstract

Cao et al. provide a comprehensive overview of signaling pathways in the pathogenesis and therapy of type 2 diabetes mellitus (T2DM) and its complications.

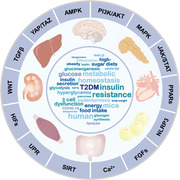

## 1 INTRODUCTION

Type 2 diabetes mellitus (T2DM), a chronic noncommunicable disease that is diagnosed by aberrant high blood glucose levels, has attracted increasing attention due to its high prevalence and enormous health burden. Currently, there are more than 537 million patients with diabetes, most of which are T2DM, and this number is projected to reach at 783 million by 2045,^1^ accompanied by a younger trend in the onset age around the world.^2^, ^3^ Beyond a straightforward rise in blood glucose, T2DM may cause a series of complications that are linked to the vascular and neural damages triggered by hyperglycemia, such as diabetic nephropathy (DN), diabetic retinopathy (DR), diabetic neuropathy, and cardiovascular disease (CVD).^3^ Of note, T2DM is emerging as an increased risk of severe COVID‐19, the recent novel coronavirus pandemic worldwide. For a long duration, T2DM and its complications have been witnessed to impose substantial effects in the quality of life and socioeconomic burden,^4^ which inspires the incredible progress in mechanism exploration and drug intervention for T2DM. However, although current antidiabetic drugs, insulin therapy, and lifestyle interventions, for example, metformin administration, carbohydrate restriction, and/or endurance exercise, have warranted decent control of T2DM progression, implementing and maintaining these changes for prolonged periods are still challenging, especially given the pervasiveness of drug side‐effects and the accessibility of calorically dense foods and sedentary lifestyle. To further develop new pharmacological strategies that could potently target pathological mechanisms and avoid side‐effects, it is still urgent and important to unceasingly disclose the mechanistic underpinnings of T2DM.

Numerous signaling pathways play essential roles in the development of T2DM and are implicated in its therapy. From a pathological view, insulin resistance (IR) and subsequent insulin deficiency due to pancreatic β cell damage are two main pathological features of T2DM, and their variable combination further contributes to the complexity of T2DM and the diversity in the patients' conditions.^1^ In addition, other pathological processes, including chronic inflammation, incretin dysregulation, hyperglucagonemia, lipolysis, central appetite dysregulation, abnormal gastric emptying, gut dysbiosis, and islet amyloid polypeptide (IAPP) deposition, are also regarded as key regulators in the pathophysiology of T2DM.^1^ The endeavors throughout the past decades have uncovered that a series of signaling pathways play important roles in controlling these pathological changes. For example, phosphoinositide 3‐kinase (PI3K)/protein kinase B (PKB, also known as AKT) signaling cascade regulates insulin response,^5^ and AMP‐activated protein kinase (AMPK) pathway prevents β cell dysfunction.^6^

From a therapeutic view, most of current glucose‐lowering drugs have been found to exert their pharmaceutical effects dependently of signaling pathways, more or less. For instance, glucagon‐like peptide‐1 (GLP‐1) receptor agonists bind to their receptors on β cells and promote insulin exocytosis by increasing intracellular Ca^2+^ levels via the protein kinase C (PKC)/cyclic adenosine monophosphate (cAMP) signaling pathway.^7^, ^8^ Recently, four categories of potential hypoglycemic drugs with new mechanisms of action have been proposed, which may stimulate insulin secretion, utilize the incretin axis, maintain hepatic glucose homeostasis, and improve insulin sensitivity, repsectively.^9^ Interestingly, these potential drugs target a number of key receptors, vital enzymes, or ion channels, such as glucokinase (GK) activators, G‐protein‐coupled receptor 40 (GPCR40), GLP‐1 receptor (GLP‐1R), and so on, which are involved in numerous signaling pathways associated with T2DM.^9^ Therefore, a better understanding of the signaling pathways is of importance for in‐depth dissecting the pathological mechanisms for T2DM and facilitating the development of targeted drugs and interventions against T2DM and its complications.

In this review, focusing on the two most important pathological features of T2DM, that is, IR and impaired β cells, align with simplified mention of other pathological changes, we elaborated the roles of signaling pathways in pathological changes of T2DM and its complications on the one hand, and expatiated the intervention mechanisms of important glucose‐lowing drugs on these signaling pathways and emphasized the important targets on the other hand.

## 2 HISTORY OF T2DM AND SIGNALING PATHWAYS

**FIGURE 1:**
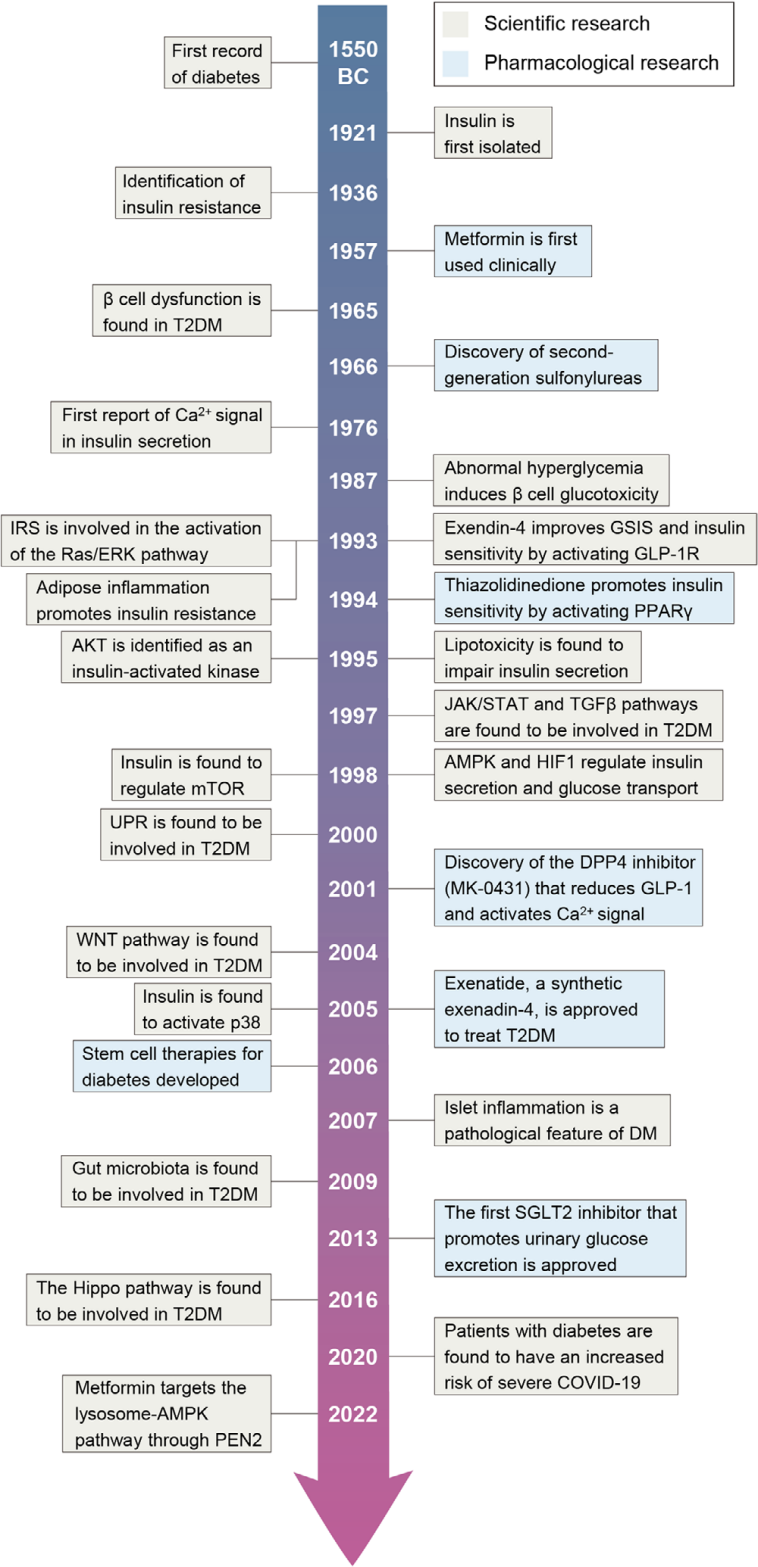
Timeline of key scientific and pharmaceutical discoveries in T2DM.

Historically, the progresses of T2DM in pathogenesis and therapy are closely related to the discovery and elucidation of signaling pathways (Figure 1). As one of the oldest diseases in human history, diabetes was first mentioned at 1552 BC,^10^ but it was not until the discovery of insulin in 1921 that understanding of the disease reached a milestone stage.^11^ The notion that serum Ca^2+^ concentration changes with insulin levels was established as early as 1930 and Ca^2+^ signaling was linked into insulin secretion in1976.^12^ Time lapsing to 1990, the intracellular transmitter and effector of insulin were discovered and the PI3K/AKT signaling was identified as the chief pathway to mediate the insulin action.^13^ Since then, mechanism of insulin action has been gradually revealed, leading to the discoveries of several classical signaling pathways related to T2DM, such as the phosphorylation of mitogen‐activated protein kinase (MAPK), AMPK, WNT, and transforming growth factor β (TGFβ) pathways.^14^ Furthermore, in recent years, several novel signaling pathways have also been linked to insulin production and action, such as fibroblast growth factors (FGFs), hypoxia‐inducible factors (HIFs), yes‐associated protein (YAP), and the unfold protein response (UPR) signaling pathways.

Continuous revelation of the pathogenesis and signaling pathways of T2DM also provides a basis for the development of drugs that target T2DM, despite the confusion of the study path. Metformin, the first‐line glucose‐lowering drug, was first synthesized in 1922, but it was not until 2022 that metformin was confirmed to exert a hypoglycemic effect by activating the presenilin enhancer 2 (PEN2)‐mediated AMPK protein^15^, ^16^ signaling pathway. Akin to metformin, sulfonylureas (SUs) were found to have hypoglycemic effects accidentally in 1942, but it was until 1979 when researchers truly found the underlying mechanism, by which acting on β cells to stimulate insulin secretion through closing K^+^ channels and activating Ca^2+^ signal.^17^ In the latest years, novel hypoglycemic drugs, such as GLP‐1 and sodium‐glucose cotransporter 2 (SGLT2) inhibitor, are effective in reducing fasting blood glucose^18^ and preventing the reabsorption of urine glucose,^19^ regardless of their unspecified mechanisms. Collectively, since these drugs have different safety, tolerability, and availability, hindering their best clinical use, it is particularly important to explore the pathogenesis and precise signaling pathways of T2DM, which would drive the identification of the targets of existing and novel drugs.

## 3 PATHOLOGICAL FEATURES OF T2DM

**FIGURE 2:**
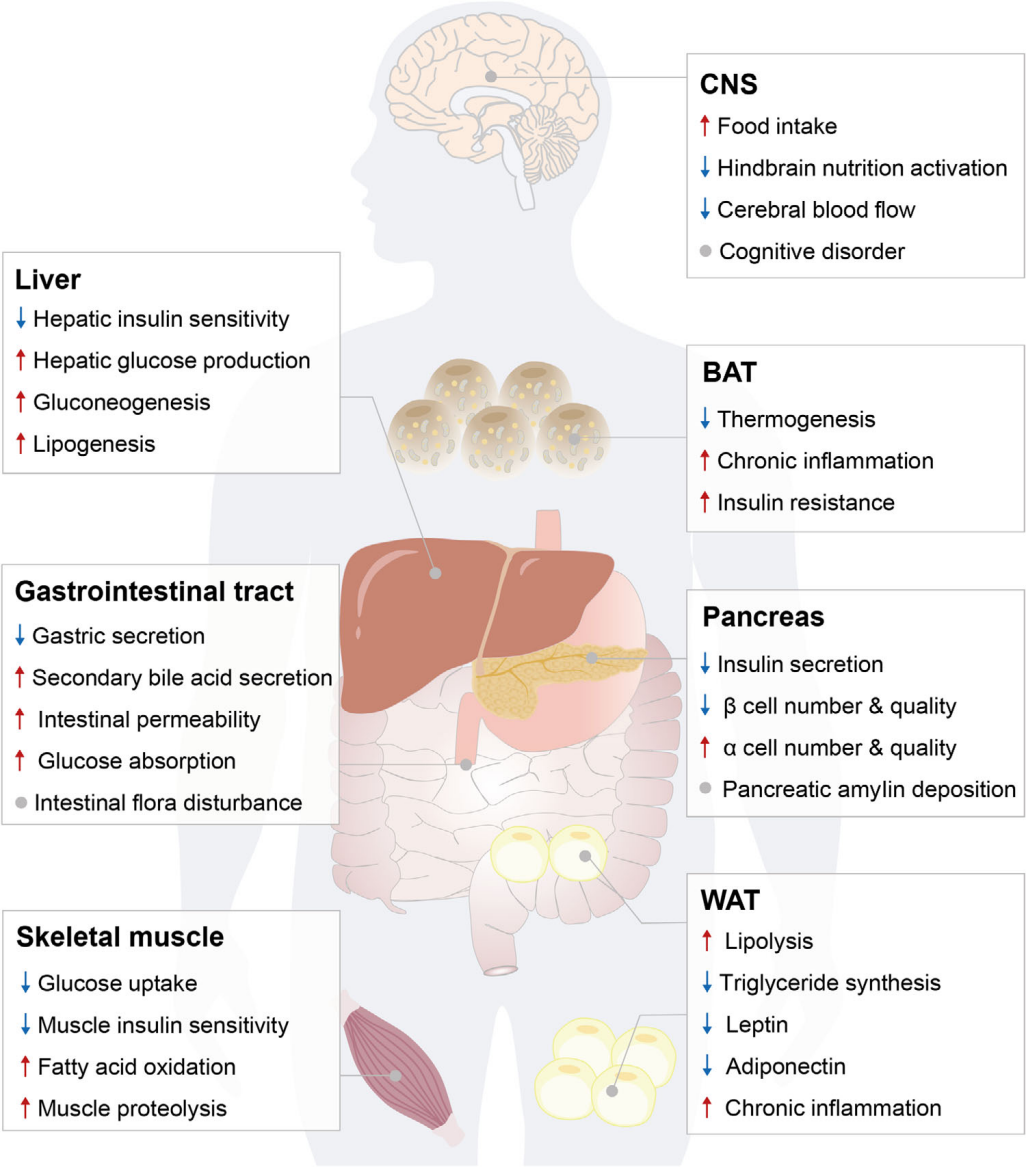
Pathological features of T2DM. T2DM is characterized by insulin resistance and β cell dysregulation, along with various pathological disturbances, which are coordinately regulated by multiple tissues and organs, including the pancreas, liver, adipose, muscle, gut, and brain. As the major characteristic of T2DM, insulin resistance occurs when the tissues become less sensitive to insulin, leading to increased glucose production and lipogenesis in the liver, enhanced lipolysis and reduced triglyceride synthesis in the white adipose tissue (WAT), and decreased glucose uptake and elevated fatty acid oxidation in the skeletal muscle, and so on. Failure of pancreatic β cells that are responsible for the production and secretion of insulin results in decreased cell number and impair insulin secretion. The vicious cycle of insulin resistance, hyperglycemia, inflammation, and so on, aggravate the onset, development, and progression of T2DM. The online resource inside this figure was quoted or modified from Scienceslide2016 plug‐in.

T2DM is conventionally featured with two pathological traits: IR and subsequent β cell dysfunction, which are the consequences of the feedback loops between disordered insulin secretion and insulin action (Figure 2).

IR, defined as the impairment of insulin sensitivity, is a predictor of T2DM and describes the failure of target organs/tissues to answer insulin stimulation.^20^ Previous studies have reported that numerous factors, including obesity, overnutrition, physical inactivity, gastrointestinal microbial disturbance, and family history of T2DM could drive IR.^21^, ^22^ Insulin is the only one hormone that actively lowers blood glucose by acting on its target organs/tissues, including the hypothalamus, liver, adipose tissue, and skeletal muscle. The hypothalamus is regarded as the major regulator of appetite, and the signaling pathways triggered by insulin, as well as leptin, play key roles in maintaining energy expenditure, glucose homeostasis and insulin sensitivity in peripheral tissues.^23^ Insulin stimulation can inspire divergent physiological responses in the peripheral tissues, including increased glycogenesis and de novo lipogenesis (DNL) but decreased gluconeogenesis in the liver,^24^ enhanced glucose uptake in the skeletal muscle and adipose tissue, suppressed lipolysis in the adipose tissue, and so on. Dysregulation of these responses, at least a part of them, is therefore to be the major consequence of IR. Additional pathological changes may be also intimately associated with IR. For example, compositional and functional alterations in gut microbiota have been observed in patients with IR and metabolic dysfunction,^25^ which may modulate cellular metabolism and energy homeostasis through homeostatic and pathogenic microbiota‐host interactions. In addition, a decrease in circulating adiponectin, an insulin‐sensitizing, anti‐inflammatory, antiatherosclerotic, and hepato‐protective factor predominantly produced by adipocytes,^26^ has been shown to extensively promote hepatic and muscular IR.^27^, ^28^, ^29^, ^30^

**FIGURE 3:**
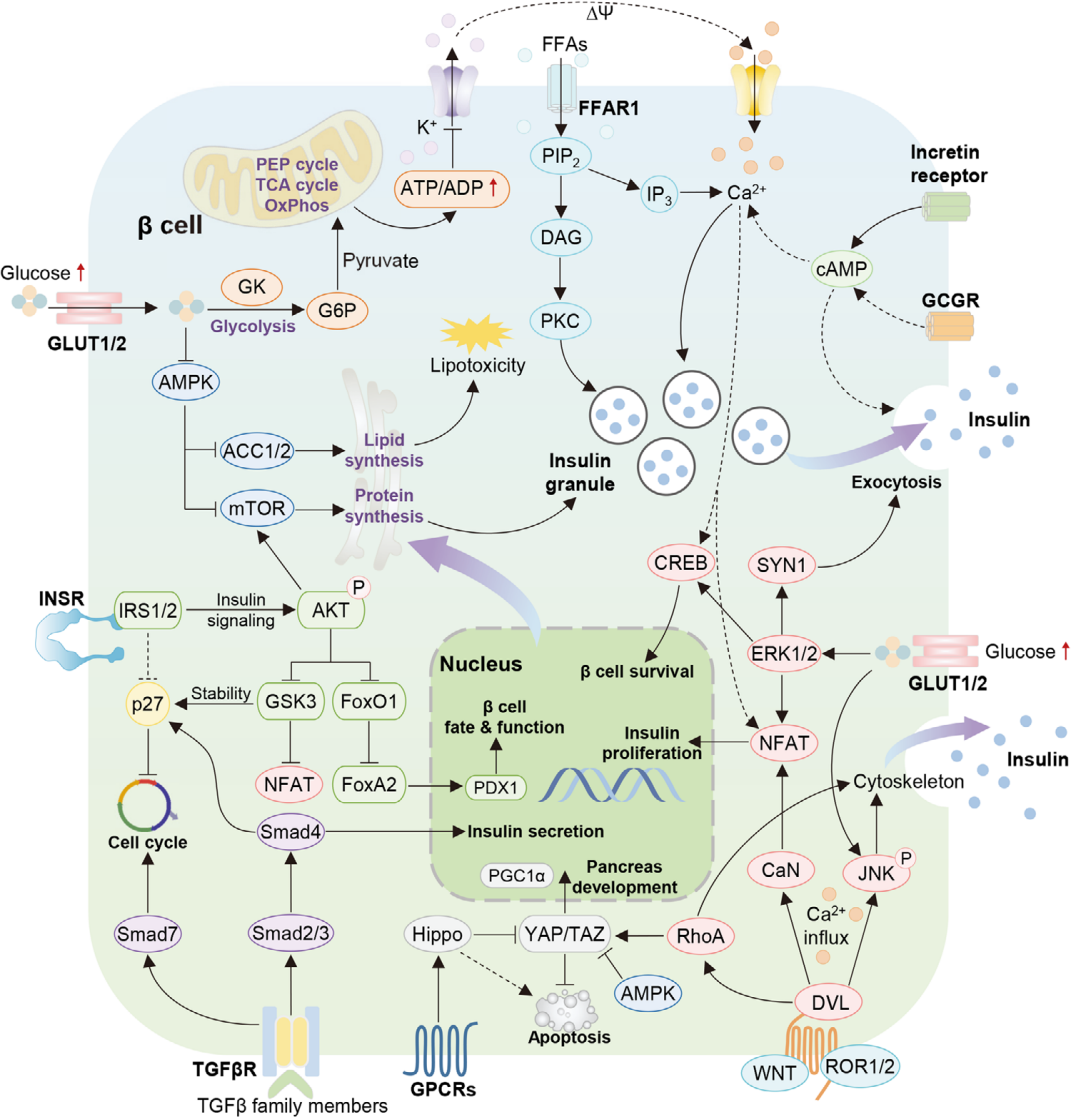
Multiple signaling pathways regulate β cell function. Elevated glucose enters the β cell via GLUT1/2 transporters to produce ATP through glycolysis in the cytoplasm and oxidative metabolism in the mitochondria, resulting in an increase in the ratio of ATP to ADP. Elevated cytoplasmic ATP leads to the close of ATP‐sensitive potassium channel, membrane depolarization, and subsequent Ca^2+^ influx, triggering the release of insulin from insulin granules. Meanwhile, multiple signaling pathways participate in mediating β cell function and insulin secretion, including AMPK, MAPK, WNT, PI3K/AKT, TGFβ, YAP/TAZ, and so on.

Pancreatic β cells function as the hub for insulin secretion (Figure 3), therefore declining β cell function due to dysregulated genetic and external factors is key to T2DM progression.^31^ β Cell dysfunction is clearly presented in the patients with hyperglycemia, but whether this trait occurs early or late in the T2DM remains controversial. The current conclusion is more inclined to that β cell function may be weakened early in T2DM progression and gradually decline as glucose tolerance deteriorates.^32^ Under normal conditions, β cells are inactive in secreting insulin during fasting period, and postprandial transient hyperglycemia can stimulate β cells to enhance glucose‐stimulated insulin secretion (GSIS), thereby increasing the blood insulin to meet the demands for lowering blood glucose. Generally, β cells are capable of producing sufficient insulin to compensate for IR and maintaining euglycemia. However, chronic exposure to excess circulating nutrients, along with the consequent changed epigenetic factors, could induce a toxic state in the pancreatic islet, resulting in β cell dysfunction and compensatory failure followed by insulin deficiency, eventually causing hyperglycemia and T2DM.^33^

Substantial evidence supports that metabolic stress could compel the progression of multiple cellular events that drive or aggravate IR and β cell dysfunction. Despite of the biological differences among divergent peripheral metabolic tissues/organs, these cellular events have notable resemblance. Roughly, they include low inflammation state,^34^, ^35^ endoplasmic reticulum (ER) stress,^36^, ^37^ mitochondrial dysfunction,^38^ lipotoxicity damage, cell death, and so on, whose interaction could cause inflammatory attack,^35^ protein turbulence,^36^, ^39^, ^40^ reactive oxygen species (ROS) accumulation,^41^ ATP deficiency,^42^, ^43^, ^44^ and ceramide overload,^45^ accelerating the pathogenesis of IR and β cell dysfunction. In particular, mitochondria serve as the prime core of glucose metabolism and ATP production in cells. Mitochondrial dysfunction, characterized as a defect in mitochondrial dynamics and thus cellular bioenergetics, is highly implicated in T2DM progression,^42^ as hyperglycemia can compel the mitochondria to enhance ROS production.^42^, ^46^ Physiologically, mitochondria can rely on its powerful plasticity of dynamic structures to restore ROS and ATP imbalances, while, as metabolic pathology proceeds, such self‐regulating mechanisms might be compromised, therefore advancing IR, β cell dysfunction and T2DM.^42^, ^47^, ^48^ Altogether, the pathological crosstalk between metabolic stresses and cellular events is a cause for the progression of IR, β cell dysfunction, and consequent T2DM.

A bevy of complicated and interdependent mechanisms have been conceptualized to dictate these cellular events, T2DM pathological features and their vicious cycles, which are primarily mediated and executed by signaling pathways. Therefore, although new identifications involving signaling pathways remain to be completely validated, it is urgent to summarize which signaling pathways and how they contribute to these pathological cellular events and T2DM.

## 4 SIGNALING PATHWAYS IN T2DM

Numerous prominent signaling pathways are involved in IR and/or β cell dysfunction, including PI3K/AKT, AMPK, MAPK, WNT, UPR, Hippo, HIFs, TGFβ, FGFs, bile acids (BAs), Ca^2+^‐related signaling pathways, and others. These signaling pathways act through their coiled interactions to enable the regulation of various biological processes regarding insulin action and production, as well as other pathophysiological modules controlling overall glucose metabolism. Adherent to this, extensive investigations have revealed that a remarkable level of dysregulations in these pathways proceed in the related cells and tissues from patients and animals with T2DM, IR, and obesity. Despite a fraction of mechanistic underpinnings are still entangled, such shifts in signaling pathways, on the one hand, relate in a large part to the metabolic stress proceeding T2DM, and on the other hand, reciprocally disrupt glucose homeostasis and thus participate in T2DM progression. Herein, we will discuss the physio‐pathological switches, roles, and mechanisms of diverse signaling pathways in T2DM development, primarily in terms of insulin action and production, along with other related biological processes.

### 4.1 PI3K/AKT pathway

**FIGURE 4:**
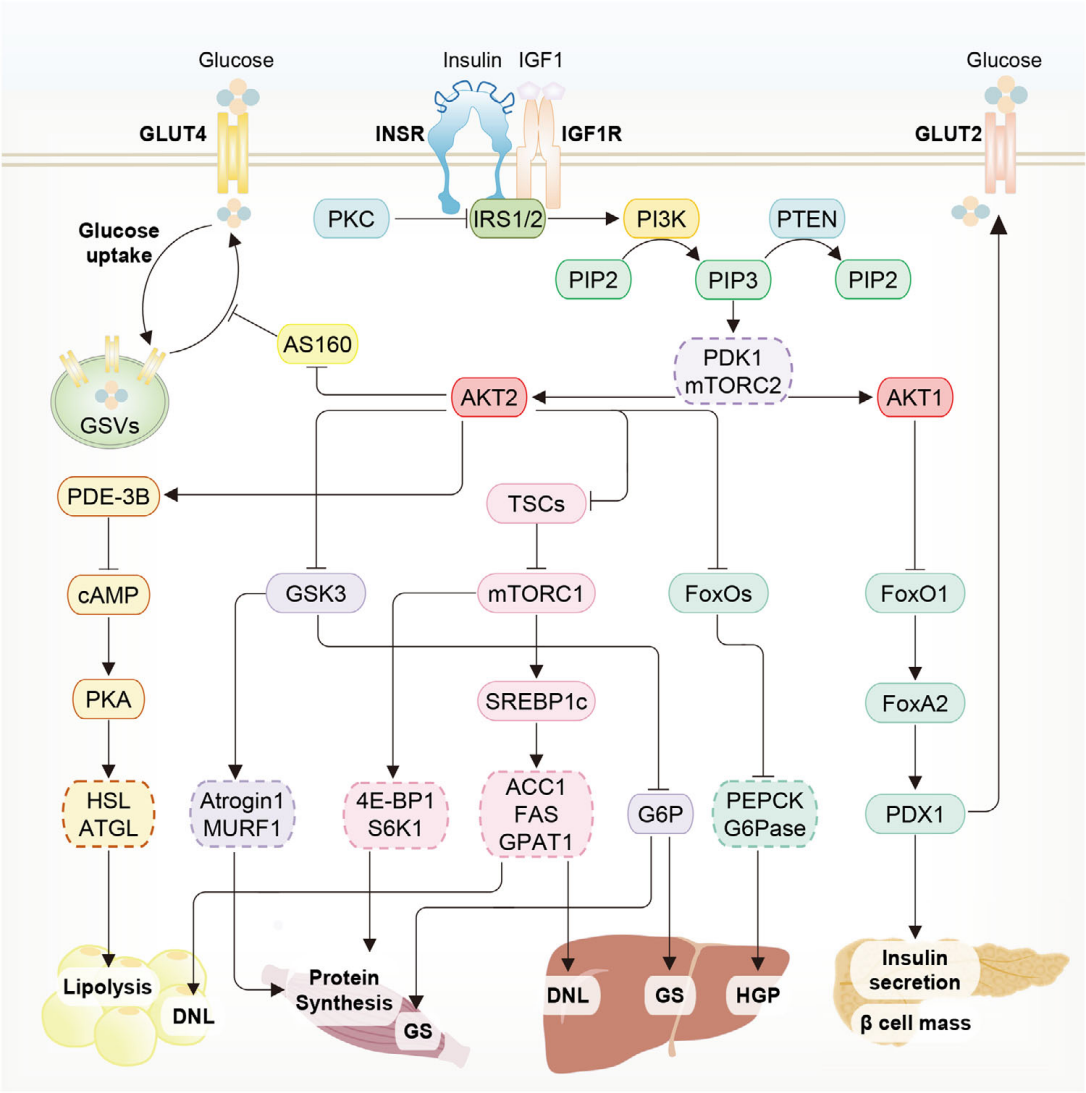
Role of the PI3K/AKT signaling pathway in different metabolic tissues. In response to insulin (or IGF1 in the skeletal muscle), the INSR (or IGFR in the skeletal muscle) is activated, causing tyrosine phosphorylation of IRS1/2. Phosphotyrosine sites on IRSs allow binding of the lipid kinase PI3K, which synthesizes PIP3 at the plasma membrane, leading to AKT phosphorylation by PDK1 and mTORC2 to fully activate the PI3K/AKT pathway. In the liver, skeletal muscle and adipose tissue, activated AKT (especially AKT2) can phosphorylate a number of substrates at Ser/Thr residues, including: (1) GSK3β, which stimulates glycogen synthesis via regulating G6P and maintaining mRNA translation by modulating Atrogin1 and MURF1; (2) TSC2, which permits activation of mTORC1 and its downstream targets 4E‐BP1, S6K1 and SREBP1c to increase protein synthesis and lipogenesis; (3) FoxOs, which decrease gluconeogenesis by suppressing gluconeogenic gene expression. In both skeletal muscle and adipose tissue, AKT inactivates AS160 to promote glucose uptake via the translocation of GSVs to the plasma membrane. In the adipose tissue, AKT also suppresses lipolysis that is mediated by PDE3B, and occurs largely through attenuation of cAMP‐stimulated events and phosphorylation of HSL and ATGL. In the pancreas, activated AKT (especially AKT1) promotes PDX1 activation and nuclear translocation by relieving FoxO1 induced FoxA2 inhibition, thereby maintaining β cell mass and insulin secretion.

Over the past decades, the PI3K/AKT pathway has been identified as the prime effector pathway in response to insulin action on the liver, adipose tissue and skeletal muscle (Figure 4). Basically, insulin binds to the extracellular domain of insulin receptor tyrosine kinase (IRTK) at cell surface,^49^, ^50^ and rapidly activates insulin receptor substrate (IRS), which then recruits and phosphorylates PI3K, and thus activate AKT.^51^, ^52^ AKTs are divided into three homologous isoforms, namely AKT1, AKT2, and AKT3.^53^ The predominantly expressed isoform in peripheral tissues is AKT2, while in pancreas, it is the AKT1, implying the tissue specificity of AKT isoforms.^53^

Although the process of insulin‐induced PI3K/AKT activation is similar, the distal steps of its activation vary across different peripheral tissues, leading to distinct biological outcomes. In the liver, the activation of PI3K/AKT pathway is responsible for decreasing hepatic glucose production (HGP), promoting glycogen synthesis, and increasing lipid biosynthesis. Activated AKT2 inhibits gluconeogenesis by phosphorylating forkhead box O families (FoxOs) and subsequently preventing the transcription of key gluconeogenic enzymes, including phosphoenolpyruvate carboxykinase (PEPCK) and glucose 6‐phosphatase (G6Pase). Meanwhile, AKT2 activation can induce the phosphorylation of glycogen synthase kinase 3 (GSK3) to promote glycogen synthesis.^54^ Additionally, AKT2 activation can promote hepatic DNL by transcriptionally enhancing the levels of several lipogenic proteins, such as acetyl‐CoA carboxylase 1 (ACC1), fatty acid synthase, and glycerol‐3‐phosphate acyltransferase 1.^50^, ^55^ Furthermore, the upstream mechanisms dictating such transcriptional regulation include the increases in mammalian target of rapamycin complex 1 (mTORC1)‐dependent sterol regulatory element binding protein (SREBP) transcription,^56^ ribosomal S6 protein kinase 1 (S6K1)‐dependent SREBP maturation,^57^ and SREBP stabilization.^58^ While in the adipose tissue and skeletal muscle, enhanced glucose uptake is a shared outcome of insulin‐induced AKT2 activation, which is mainly related to an increase in the density of glucose transporter 4 (GLUT4) in the plasma membrane.^59^, ^60^ Moreover, AKT2 activation in the adipose tissue can promote DNL via the mechanisms similar to those in the liver and suppress the lipolysis through multiple downstream mechanisms. These mechanisms involve the suppression of cAMP‐dependent protein kinase A (PKA) activity^61^ and the regulation of lipolytic regulatory proteins, such as mTORC1,^62^ S6K1,^63^ protein phosphatase 1 (PPI),^64^ protein phosphatase 2A (PP2A),^65^ and interferon regulatory factor 4 (IRF4).^66^ In summary, the well‐proceeding of these downstream branches of AKT confers the peripheral insulin action with the power to reduce the blood glucose, or in other words, maintains insulin sensitivity, thereby contributing to glucose homeostasis.

Consistent with its protective effect on insulin sensitivity, the PI3K/AKT pathway is usually impaired in the insulin‐resistant tissues, thus disrupting the key metabolic actions of insulin.^38^, ^67^ Actually, AKT2 knockout mice usually exhibit T2DM phenotype with IR and glucose intolerance.^46^, ^47^ The decreased ability to activate PI3K/AKT pathway could derive from several pathological facets related to IR and T2DM, including the mutation of this pathway itself, dysregulation of some regulator proteins or other signaling pathways, lipid accumulation or alteration, reduced circulating adiponectin, and enhanced inflammation state. In T2DM, these factors together with the impaired AKT pathway, control IR progression and thus systemic glucose state in a complicated way.

Alterations of both the upstream and downstream components in the PI3K/AKT pathway itself have been found to aggravate peripheral IR. For example, insulin receptor (INSR) with a single mutation at leucine 973 significantly attenuated insulin/PI3K/AKT activation in the liver and white adipose tissue (WAT), impaired systemic insulin sensitivity, and thus decreased insulin‐induced glucose uptake.^68^ In parallel, mice with knockout of insulin and/or insulin like growth factor 1 (IGF1) receptors developed IR, glucose intolerance, and islet hyperplasia with hyperinsulinemia, accompanied with increased lipolysis and adipocyte apoptosis.^69^ Meanwhile, fat‐specific disruption of the downstream effectors of AKT pathway, FoxOs, can result in a reversal of IR in the liver, an exacerbation of hyperinsulinemia, but a maintenance of normal glucose tolerance.^70^

Dysregulation of certain regulators might also compromise insulin sensitivity and glucose homeostasis through altering AKT pathway. For instance, phosphatase and tensin homolog (PTEN), a negative regulator that induces IR by converting PIP3 back to PIP2 via dephosphorylation, is often overexpressed in T2DM patients,^71^ contributing to the termination of PI3K/AKT2 signaling network and the progression of IR.^72^, ^73^ Liver‐specific deletion of sirtuin 1 (SIRT1) has also been pointed out to cause ROS accumulation and thus impair AKT signaling, eventually inducing IR, hepatic glucose overproduction and chronic hyperglycemia.^74^ Consistently, the activation of ApoM/S1P complex, which also activates the AKT pathway, can prevent IR progression through upregulating SIRT1.^75^ More recently, it was shown that TGFβ1 stimulated clone 22 D4 (TSC22D4) is a novel interaction partner for AKT, and its dysregulation is able to disrupt insulin sensitivity and glucose disposal in mice.^76^

**FIGURE 5:**
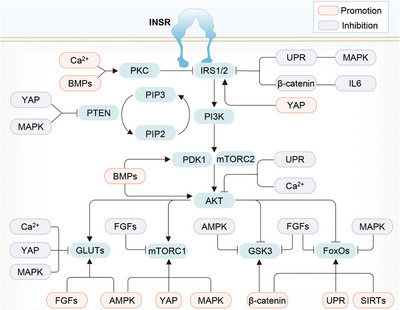
Crosstalk between PI3K/AKT and various pathways. The PI3K/AKT signaling inactivator PKC is promoted by the BMP family via the TGFβ pathway and Ca^2+^ overloading, while PTEN is inhibited by MAPK and YAP pathways. IRS2 is activated by the YAP pathway, but repressed by multiple signals, including MAPK, UPR, and WNT pathways, and proteins, such as IL‐6 and IKKβ. Both AKT and PDK1, are activated by BMPs, while AKT is in turn dephosphorylated by the UPR pathway and Ca^2+^ overloading. The AKT downstream GSK3β can be activated by the UPR pathway, but blocked by the AMPK signaling and FGF family; FoxOs are also stimulated by the UPR pathway in line with β‐catenin and SIRTs, while inhibited by the MAPK signaling and FGF family; mTORC1 can be motivated by MAPK, AMPK, and YAP pathways, but repressed by FGFs pathways. The glucose transporter GLUTs expression and membrane translocation can be improved by the AMPK signaling and FGF family, while inhibited by the MAPK and YAP pathways, as well as Ca^2+^ overloading.

It is well known that lipid metabolism and chronic inflammation could modulate AKT‐associated insulin sensitivity and IR. Diacylglycerol accumulation in the liver impairs the AKT activity through PKC,^77^, ^78^, ^79^, ^80^ an inhibitor of the PI3K/AKT2 pathway.^81^ Similarly, under T2DM conditions, exosomes with altered lipid composition can be produced by the intestine and taken up by the macrophages and hepatocytes to repress the hepatic insulin/PI3K/AKT signaling pathway.^82^ Reduced circulating adiponectin,^29^, ^30^ such as globular adiponectin (gAcrp30), could also cause hepatic and muscular IR by increasing ectopic lipid storage in these organs.^83^ Furthermore, enhanced inflammation state, caused by certain dysregulated inflammatory modulators, such as protease‐activated receptor 2 (PAR2),^84^ and neurite outgrowth inhibitor (Nogo),^85^ have also been linked to impaired AKT activation and thus peripheral IR. Besides, numerous microRNAs (miRNAs),^86^ for example, miR‐26a, can interfere with many proximal PI3K/AKT pathway components to regulate insulin sensitivity and glucose metabolism.^87^ In addition, a wide scope of signaling pathways have been characterized to modulate the consequence of insulin‐triggered AKT activation through directly regulating AKT or indirectly affecting AKT upstream and downstream branches, thereby influencing the progression of IR and T2DM (Figure 5). These signaling pathways, as well as their interplay with the PI3K/AKT cascade, will be introduced in the below sections.

In the pancreatic islet, PI3K/AKT1 activation can increase β cell mass and stimulate insulin production, indicating another link between the dysregulation of PI3K/AKT pathway and T2DM progression. Increasing evidence indicates that β cell‐specific overexpression or constitutive activation of AKT1, as well as knockout of PTEN,^88^ FoxO1,^89^ tuberous sclerosis complexes (TSCs)^90^ or mTORC1,^91^ are able to increase β cell mass, proliferation, neogenesis, and cell size, thereby improving glucose tolerance. On the contrary, specific knockout of 3‐phosphoinositol dependent protein kinase 1 (PDK1),^92^ IRS2,^93^, ^94^ INSR,^95^ IGF1,^96^ or S6K1,^97^ can repress AKT1^98^ signaling transduction, leading to the decreases in insulin content and secretion, β‐cell mass and proliferation, and glucose tolerance, and eventually facilitating the development of hyperglycemia and T2DM. Mechanistically, the PI3K/AKT1 pathway relies on the transcriptional factor pancreatic and duodenal homeobox 1 (PDX1) to control β cell differentiation, function, survival and proliferation.^53^ PDX1 controls the expression of multiple key genes for β cell fate, such as insulin (Ins1 and Ins2), neurogenin 3 (Ngn3), SRY‐box transcription factor 9 (Sox9), v‐mafmusculoaponeurotic fibrosarcomaoncogene homolog A (MafA), Glut2, GK (Gck), iapp, cyclin D1/2 (Ccnd1/2), and transient receptor potential canonical 3/6 (Trpc3/6).^99^ All in all, as the core signaling pathway downstream of peripheral insulin action or a potent regulator of β cell function, the PI3K/AKT pathway holds a great power in the regulation of T2DM progression, and a notable potential to be a drug target for effectively treating T2DM.

### 4.2 AMPK pathway

**FIGURE 6:**
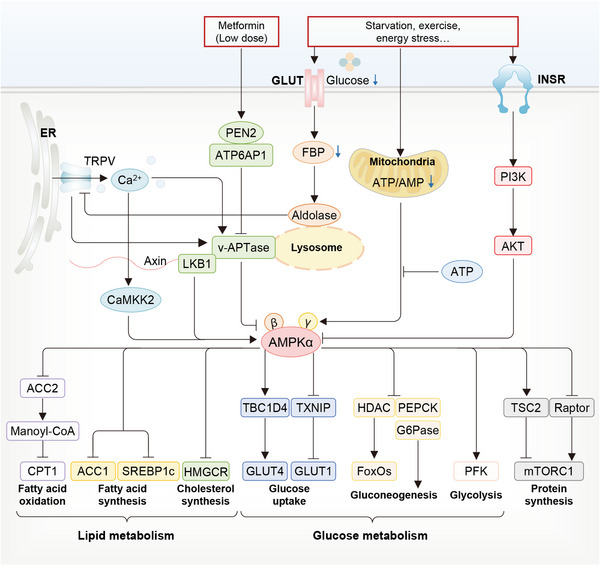
The AMPK pathway regulates glycolipid metabolism in T2DM. Under conditions of energy requirement, such as starvation, exercise and OS, the decrease of ATP/AMP ratio activates AMPK directly. Meanwhile, low glucose decreases FBP level, which is sensed by aldolase. Reduced FBP only binds to a small number of aldolases, the remaining which occupy TRPV. Aldolase strongly interacts with TRPV channels and blocks Ca^2+^ release, suppressing v‐ATPase activity and stimulating AMPK activity. Metformin interacts with PEN2 and inhibits v‐ATPase activity, thereby activating AMPK. Besides, obesity‐induced hyperinsulinemia activates protein kinases to suppress AMPK activity. Upon activation, AMPK acts on multiple downstream targets to increase ATP generation and decrease ATP consumption by inhibiting anabolic processes and increasing catabolic processes, subsequently regulating a series of downstream targets to affect glucose and lipid metabolism.

The AMPK pathway is famous for its critical role in sensing cellular energy status (Figure 6).^100^ AMPK can be canonically activated by the increasing AMP and/or ADP along with declining ATP through the upstream kinase liver kinase B1 (LKB1)‐dependent Thr172 phosphorylation, or noncanonically activated by other stimulations through several recently described pathways that are independent of AMP/ADP, including those related to the lysosome, mitochondrion, Ca^2+^/calmodulin‐dependent protein kinase kinase 2 (CaMKK2) and TGFβ‐activated kinase 1 (TAK1).^101^, ^102^, ^103^ Owing to these mechanisms, AMPK can sense the availability of glucose, glycogen, FAs, Ca^2+^, leptin, and adiponectin, and the damage to lysosomes and nuclear DNA, as well as the stimulation of multiple drugs.^15^, ^102^ Basically, activated AMPK pathway is able to endorse ATP‐producing catabolic pathways via phosphorylating and activating certain proteins, while curb energy consumption via phosphorylating and inactivating proteins involved in anabolic (biosynthetic) pathways, thereby balancing cellular metabolism and functions.^102^, ^104^ In this regard, the downstream events of the AMPK pathway encompass: carbohydrate or glucose metabolism, FA and cholesterol metabolism, protein synthesis, the counteracting effects on mTORCs, mitochondrial biogenesis, mitophagy, and autophagy.^100^, ^105^ Such potent effects of the AMPK pathway in cellular metabolism and functions are prone to endow it with an incredible significance in the peripheral insulin action, glucose uptake, nutrient intake, lipid metabolism, inflammation, insulin secretion, and thus systematic homeostasis of glucose and lipids, warranting the vigorous attention of its therapeutic potential in the area of T2DM.^104^

Degrading of AMPK activity is widely observed in the skeletal muscle from mice and humans with obesity and T2DM, probably due to that high glucose could inhibit the AMPK activity by breaking the active LKB1 complex,^106^ activating the protein phosphatase PP2A,^107^ upregulating the phosphatase PH domain and leucine rich repeat protein phosphatase 2 (PHLPP2),^108^ and inducing ubiquitination degradation of AMPK subunits.^109^, ^110^ AMPK activation has been found to exert constructive effects on the glucose uptake of skeletal muscle, and regarding this, the deficiency of muscular AMPK activity might compromise the whole‐body glucose homeostasis during T2DM. In detail, the activation of AMPK increases glucose uptake through the mechanisms by which involve the inhibited sequestering of GLUT4 to Golgi apparatus, the enhancement of GLUT4 translocation to plasma membrane, and the upregulation of GLUT4 expression.^103^ In the cells that hinge on GLUT1, AMPK also boosts their glucose uptake through promoting the translocation of GLUT1 to the plasma membrane and increasing GLUT1 expression.^111^ Since glucose uptake is a key step in the downstream of insulin action, such physiological supportive effect of AMPK in glucose uptake might bypass the PI3K/AKT pathway to link AMPK activation with IR inhibition.^112^ In fact, muscle contraction, hypoxia and adiponectin stimulation have been found to initiate GLUT4 translocation via the activation of AMPK in the skeletal muscle, which might counteract muscular IR.^20^ Supporting this, multiple drugs or natural products that activate AMPK can improve IR and glucose homeostasis in the mice and patients with obesity or T2DM, including metformin,^15^ O304,^113^ MK‐8722,^114^ Canagliflozin,^115^ and PF‐739.^116^ Interestingly, although AMPK does not directly regulate the insulin/AKT pathway, the ATK pathway has instead been identified to inhibit AMPK activation,^117^ which may complicate the relationship between the AMPK pathway and insulin action. Collectively, identification of accurate alterations, detailed mechanistic cues, and pharmaceutical activators regarding the skeletal muscle‐specific AMPK pathway might confer a basis for the advancement of T2DM mechanistic investigation and drugs intervention.

In recent perspectives, hepatic AMPK activation appears to indirectly inhibit hepatic gluconeogenesis, refreshing the previous understanding of its direct effect on HGP.^103^ Nevertheless, genetically or pharmacologically induced activation of AMPK in hepatocytes can still indirectly suppress gluconeogenesis, alleviate the liver IR, lower HGP, and improve glucose parameters in the human and animals with obesity and T2DM.^103^, ^118^ These effects are primarily mediated through the inhibition of DNL, the depression of inflammatory makers, and the promotion of mitochondrial function in the liver and other organs.^119^ Hence, it is of importance for future study to deepen our knowledge and understanding of the exact role of AMPK in hepatic glucose metabolism.

The AMPK pathway can also participate in T2DM progression by controlling a wide scope of metabolic processes that are not directly linked to peripheral IR. First, hypothalamic AMPK activation under ghrelin or low glucose stimulation can improve appetite via inhibiting ACC1,^120^ and activating autophagy^121^ and p21‐activated kinases,^122^ to enhance energy supply. Conversely, AMPK inactivation in response to leptin and insulin suppresses appetite, preventing obesity and T2DM. Second, AMPK activation is able to widely promote FA oxidation in the skeletal muscle and liver through inhibiting the phosphorylation of ACC1/2, thus indirectly amending hyperinsulinemia, glucose intolerance and IR.^119^ Meanwhile, the suppression of cholesterol synthesis also responds to AMPK activation, in which involves the inhibition of 3‐hydroxy‐3‐methylglutaryl (HMG) coenzyme A (CoA) reductase (HMGCR).^123^ Third, AMPK activation can stimulate glycolysis through the phosphorylation and activation of phosphofructokinase (PFK), and repress the glucose storage by inhibiting multiple isoforms of glycogen synthase (GS).^105^, ^124^ Forth, the activation of AMPK pathway has been linked to the depression of inflammation in macrophages, adipose tissue, liver and skeletal muscle, thereby ameliorating systemic IR and improving glucose homeostasis.^125^ Finally, the important role of AMPK activation in maintaining mitochondrial homeostasis may also prime its indirect role in ensuring metabolic efficiency of cells and tissues.^105^ In line with this, adipocyte‐specific deficiency of AMPK in mice worsens HFD‐induced systemic IR through disrupting mitochondrial integrity in adipocytes, in terms of the mitochondrial function, structure, and markers of mitophagy.^126^

In the pancreatic β cells, the AMPK/mTOR pathway may affect T2DM progression by modulating β cell mass and insulin secretion.^127^ It has been reported that the switch from mTORC1 to AMPK is the basis for the growth of β cells during embryonic and early postnatal life, including promoting β cell mitochondrial biogenesis and functional maturation of oxidative metabolism.^128^ In detail, the AMPK pathway represses mTORC1 activation to confer β cells with functional maturation, and therefore decreased AMPK activation in diabetic islets may be prime for enhanced mTORC1 signaling.^129^ Despite physiological mTORC1 activation has positive effects on β cell survival, proliferation, and homeostasis,^130^ sustained mTORC1 activation in the cases of overnutrition or hyperinsulinemia, conversely leads to β cell failure and impaired GSIS, which can be inversed by a short‐term inhibition on mTORC1 activation.^131^ In addition, it has been showed that abnormally increased glycolytic metabolites caused by chronic hyperglycemia can engender an inhibition of AMPK but an activation of mTORC1 to reprogram metabolic gene expression, weaken mitochondrial glucose metabolism and ultimately impair GSIS.^132^ In summary, since switches between mTORC1 and AMPK underlie β‐cell metabolic plasticity, the AMPK/mTOR pathway might play prestigious roles than previous thought in the β cell function and T2DM progression.

Although the AMPK pathway has been regarded as a potent target for T2DM treatment, its effect on GSIS is still entangled. For instance, LKB1, the key upstream activator of AMPK, has debatable roles in insulin secretion. On the one hand, LKB1 deficiency in β cells could promote insulin secretion by elevating ACC1 activity and plasma membrane excitability.^133^, ^134^ On the other hand, the absence of LKB1/AMPK activity also promotes the mitochondrial impairment that compromises GSIS. Notably, a recent study reported that AMPK activation influences GSIS in β cells in the manners dependent of action duration and glucose concentration.^135^ Specifically, drug activation of AMPK primes GSIS in a short duration, while a long‐term AMPK activation represses insulin secretion.^135^ Meanwhile, only a high level of glucose action can potentiate insulin secretion.^135^ Additionally, the promoting effect of AMPK on GSIS has been recently reported. It has shown that β cell‐specific deletion of AMPK increases the levels of miR‐125b‐5p, which could subsequently impair GSIS in both MIN6 cells and human islets.^136^ As well, silencing of the metal‐dependent protein phosphatase 1E (PPM1E), the most markedly downregulated protein phosphatase in T2DM patients’ islets, can promote GSIS through increasing the phosphorylation of CaMKII, AMPK, and ACC.^137^ Altogether, these conflicting results warrant further investigations to provide more reliable information on the role of AMPK in T2DM and its clinical application.

### 4.3 MAPK pathway

**FIGURE 7:**
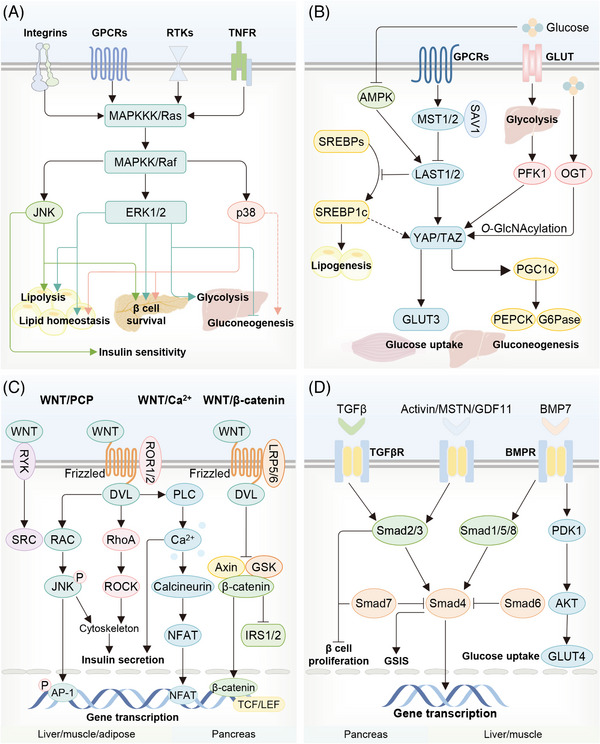
Involvement of MAPK, YAP, Wnt, and TGFβ pathways in T2DM. (A) The classic MAPKKK–MAPKK–MAPK or Raf–Ras–MAPK signaling pathway can be activated by various metabolic signals. Upon being activated, MAPKs, comprising of three subtypes (ERK1/2, JNK1/2/3, and p38 family), induce diverse metabolic responses, including chronic inflammation state, insulin resistance, and altered gluconeogenesis in metabolic tissues, as well as improved insulin secretion and survival of pancreatic β cells. (B) The Hippo pathway is mainly composed of MST1/2 and LATS1/2. Active MST1/2 phosphorylates LATS1/2, which in turn phosphorylates YAP/TAZ and thus inhibits YAP/TAZ activity. Metabolites and hormones could act through GPCRs to regulate the Hippo pathway and YAP/TAZ. Deficient glucose metabolism decreases YAP/TAZ activity and prevents the formation of YAP–TEAD complex. Subsequently, YAP/TAZ could regulate various glycolipid metabolism processes in different metabolic organs. (C) WNT pathway encompasses three different branches, including WNT/β‐catenin, WNT/PCP, and WNT/Ca^2+^ pathways. Under metabolic stress, WNT/β‐catenin pathway could aggravate hepatic insulin resistance and enhance hepatic glucogenesis. WNT/PCP pathway increases hepatic insulin resistance via activating JNK pathway, whereas activated WNT10b/β‐catenin and WNT/PCP pathways improve the insulin sensitivity of skeletal muscle. Moreover, WNT/Ca^2+^ pathway could regulate insulin secretion in β cell. (D) The TGFβ pathway could influence T2DM development by affecting the function of pancreatic islet as well as the insulin signaling of nonislet tissues. TGFβ inhibits β cell proliferation, facilitates the generation of β cell, and enhances GSIS. Moreover, different WNT ligands have specific effects in adipose insulin resistance and hepatic gluconeogenesis.

The MAPK signaling pathways contain three major subclasses, namely the extracellular signal‐regulated kinases 1/2 (ERK1/2), c‐Jun N‐terminal kinases (JNKs), and p38 family (Figure 7A).^138^, ^139^, ^140^ Multiple stimuli, such as hormones, growth factors, and TGFβ‐related agents, have been identified to activate MAPK pathways via a dedicated three‐tiered protein kinase cascade that is comprised of a MAPK kinase kinase (MAPKKK), a MAPK kinase (MAPKK), and the MAPK.^141^ Activated MAPK pathways indorse selective phosphorylation of transcriptional factors, for example, nuclear factor of activated T cells (NFAT), activator protein 1 (AP‐1), and C/EBP‐homologous protein (CHOP)/DNA damage‐inducing protein 34 (GADD34), as well as protein kinases, for example, ribosomal s6 kinases (RSKS) and eukaryotic initiation factor 4E (eIF4E), thus controlling gene transcription and signaling transduction.^141^ As such, they are capable of connecting extracellular stimuli to cellular events such as proliferation, inflammation, differentiation and apoptosis. Accumulating evidence suggests that MAPK pathways are altered in several metabolic tissues during T2DM progression and play significant roles in peripheral IR and β cell fate, despite of the discrepancies among the three subclasses.^142^

It appears that ERK1/2, JNKs, and p38s pathways are all basically activated in the liver of mice and humans with metabolic stress, and contribute to impaired hepatic insulin sensitivity and glucose metabolism, exacerbating the progression of T2DM. Hepatic ERK1/2 activities have been found to be increased in both genetic and diet‐induced obesity mouse models,^143^ triggering overall IR and impaired glucose homeostasis, while obese mice with decreased hepatic ERK1/2 showed better systemic insulin and glucose tolerance. Mechanistically, activation of ERK1/2 could participate in a range of biological activities that function individually or interdependently in aggravating IR progression. These biological activities roughly include the serine phosphorylation of IRS proteins,^143^ the connection between the CBA stimulation and impaired hepatic glucose metabolism,^144^, ^145^ the negative effect of hepatocyte‐derived fibrinogen‐related protein 1 (HFREP1) in hepatic insulin sensitivity,^146^ the positive feedback between cytokine secretion of macrophages and IR of hepatocytes,^147^ and the gluconeogenic response of FGF21 stimulation on the liver.^148^ Additionally, the protective effect of serum‐ and glucocorticoid‐regulated kinase 1 (SGK1) in hepatic insulin sensitivity relies on the inhibition of ERK1/2 activity.^149^

Similar to ERK1/2, hepatic JNKs are also activated by obesity. Hepatic activation of JNK is believed to reduce the expression of peroxisome proliferator‐activated receptor α (PPARα) target genes, for example, *FGF21*, to block FA oxidation and thus aggravate IR.^150^ Correspondingly, liver‐specific deficiency of functional JNKs in mice ameliorates diet‐induced IR and hyperglycemia.^151^ Akin to ERK1/2 and JNKs, hepatic activation of p38 family is also linked to glucose intolerance and hyperinsulinemia. Supporting this, either expression of dominant‐negative p38α or inhibition of p38α in the liver could decrease fasting insulin levels and recuperate glucose tolerance in obese mice.^152^ In detail, p38α activation induces the serine phosphorylation of IRS1 to compromise hepatic insulin sensitivity^152^ and upregulates gluconeogenesis by blunting the activation of AMPK pathway^153^ and instigating the expression of genes including *CREB*, *C/EBPα*, *PPARα*, and *PGC1α*.^142^ However, one important study once noted that hepatic p38 activity is reduced in the livers of obese mice, which is contrast to the previous findings, and hepatic activation of p38 can attenuate ER stress and reset glycemia in diabetic mice by enhancing nuclear translocation of X‐box binding protein 1s (XBP1s).^154^ Future studies are hence required for identifying the real role of p38 signaling pathways in hepatic glucose metabolism.

Multiple studies delineated that JNK1 is usually activated in the adipose tissue of high‐fat diet (HFD)‐fed mice to aggravate IR. Inversely, adipocyte‐specific deletion of JNK1^155^, ^156^ or JNK interacting protein 1 (JIP1),^157^ a key protein activating JNK, could restore insulin action in the adipose tissue with metabolic stress. The contribution of JNKs pathway in establishing adipocyte IR springs from its promotive role in the inflammation of adipose tissue. Activated JNKs can trigger the secretion of HMGB1, a proinflammatory adipocytokine, and thus promote WAT inflammation and IR in obese patients.^158^ Meanwhile, macrophage‐specific JNK deficiency was shown to reduce the polarization of proinflammatory macrophages in the adipose tissue.^155^ Additionally, several studies unconcealed that ERK1/2 is also abnormally activated in the diabetic adipose tissue to facilitate adipocyte IR by inducing adipogenesis^159^ and local inflammation state,^160^, ^161^ despite of the obscure molecular mechanisms.

In the skeletal muscle, MAPK pathways are also deviant in obese or diabetic mice,^142^, ^162^ suggesting their potential roles in modulating muscular IR and glucose metabolism.^163^ In fact, ERK1/2 pathway has been observed to negatively control the action of GS in myotubes in a manner independent of GSK3.^164^ The abnormal activation of JNK1 in the skeletal muscle was also detected in the HFD‐fed mice, and muscular blocking of JNK significantly reduced obesity‐induced hyperglycemia by halting inflammation and IR, as well as enhancing glucose uptake of skeletal muscle.^165^ Basically, mice with increased activity of overall p38 in the skeletal muscle could prevent the development of diet‐induced obesity and IR by enhancing miR‐21 expression and repressing PTEN expression.^166^ It is of interest to note that distinct p38 isoforms might have differential metabolic functions in the skeletal muscle. For example, p38α/β were apprised to advocate the inflammatory state by initiating inflammatory cytokine expression and infiltration of proinflammatory macrophages,^167^ while p38γ was demonstrated to elevate basal glucose uptake but decrease contraction‐stimulated glucose uptake, partially by changing the expression of GLUT4 in skeletal muscle.^163^ Meanwhile, it appears that the p38β is responsible for the major catabolic action of p38 family by affecting the C/EBPβ activity.^168^

In the pancreas, the activity of ERK1/2 is also obviously upregulated by hyperglycemia,^169^ thereby promoting GSIS and survival of pancreatic islets during T2DM progression. Indeed, ERK1/2 activation in the β cells is sensitive to the glucose and GLP‐1 action,^170^ and further upregulates the transcription of genes related to insulin production and secretion^171^, ^172^ by adjusting the formation of transcriptional complex composed of NFAT and its partners.^173^ In parallel, ERK1/2 may contribute to the exocytosis of insulin granules via inducing phosphorylation of synapsin I (a key protein in exocytosis),^174^ and the first phase of GSIS showed a reduction of 40% in mice that ERK1 and ERK2 are inhibited simultaneously.^175^ Moreover, many in vitro and in vivo studies^176^ also indicate that ERK1 is indispensable for the glucose‐induced activation of genes responsible for β cell survival, such as mitogen‐ and stress‐activated kinase 1 (MSK1) and CREB.^175^ However, other MAPK pathways might differentially affect the biology of β cells. One significant instance is that suppression of p38 and JNK pathways is essential for metformin to upregulate the expression of pancreatic aquaporin 7 (AQP7) and subsequently induce glycerol influx and insulin secretion of β cells in T2DM.^177^ Therefore, there is a calling for more investigations about the accurate roles of different MAPK pathways in the pancreas biology.

What's more, sustained activation of MAPK/ERK signal transduction in hypothalamus has also been uncovered to be key to the antidiabetic action of intracerebroventricular injected FGF1 and subsequent remission of hyperglycemia T2DM rodents.^178^ Taken together, one interesting aspect that emerged from the existing findings of MAPK pathways in T2DM is that targeting MAPK signaling potentially broaden the scope of antidiabetic interventions.

### 4.4 WNT pathway

The WNT pathways are classified into two major groups, β‐catenin‐dependent (canonical) or β‐catenin‐independent (noncanonical) (Figure 7C).^179^ The nuclear translocation of β‐catenin is key to activating canonical WNT pathway, where it binds to the transcription factor T‐cell factor/lymphoid enhancer‐binding factor (TCF/LEF) and consequently controls the transcription of target genes.^179^ The noncanonical pathways are subdivided into the WNT/Ca^2+^ pathway and WNT/planar cell polarity (PCP) pathway.^180^ Activated WNT/Ca^2+^ pathway enhances Ca^2+^ influx and initiates various signaling pathways that phosphorylate RORα and induce nuclear translocation of NFAT and nemo like kinase.^180^ The WNT/PCP pathway works in both dishevelled (DVL)‐independent and ‐dependent ways: the binding of WNT to the receptor like tyrosine kinase (RYK) is capable of activating protein tyrosine kinase (SRC) independently of DVL, while its binding to the ROR1/2‐Fzd complex can activate DVL, a central mediator of WNT/PCP pathway, and then trigger the activation of Ras‐related C3 botulinum toxin substrate 1 (RAC1), Ras homolog gene family (RhoA), and cell division cycle 42 (CDC42), thereby governing the cytoskeleton remodeling and the activation of AP‐1 and NFAT.^181^, ^182^ Additionally, some novel noncanonical WNT pathways, such as WNT/mTOR, WNT/YAP/TAZ, WNT/LRP5/mTOR/AKT and WNT/Hippo, have been delineated.^182^, ^183^ It is warranted that aberrant WNT pathways play important roles in multiple pathological processes, including IR and β cell dysfunction,^184^, ^185^, ^186^ and are causative to the progression of T2DM,^185^, ^187^ yielding a latent therapeutic strategy for treating this disease.^184^, ^188^

Dysregulated WNT pathways has been implicated in hepatic IR. It has shown that overexpression of β‐catenin is correlated with a rise in fasting glucose concentrations, while specific knock‐out of β‐catenin in the liver is sufficient to improve hepatic insulin sensitivity and decrease blood glucose concentrations in obese mice.^189^ Mechanistically, decreased phosphorylation of hepatic IRS1/2 and GSK3β may mediate the inhibitory effect of WNT/β‐catenin pathway in insulin sensitivity. Meanwhile, the abundant FoxO1 nuclear accumulation connects WNT/β‐catenin activation with hepatic glucogenesis. Consistent with these findings, mice with a knockdown of LDL receptor‐related protein 6 (LRP6), a WNT coreceptor, were resistant to HFD‐induced hyperglycemia and hepatic IR, probably due to the enhanced transcription of leptin receptor.^189^ Furthermore, aberrant expression of the key effector of WNT/β‐catenin pathway, transcription factor 7 like 2 (TCF7L2), has been witnessed to induce the transcription of gluconeogenic enzymes (e.g., FBP1, PCK1, and G6Pase) and insulin signaling proteins (e.g., IRS1/2 and AKT2).^190^, ^191^ Intriguingly, a number of gene *TCF7L2* variants are correlated with the susceptibility of T2DM, which primes them as effective predictors of T2DM risk.^192^ Hepatic activation of WNT/PCP pathway was also observed to trigger the serine phosphorylation of IRS1 via activating the JNK signaling.^193^ In addition, intercellular and interorgan communications might contribute to the hepatic consequences of WNT. For example, secreted frizzled‐related protein 4 (sFRP4), an adipokine with elevated expression in obese WAT, can function as a WNT antagonist and promote hepatic DNL and IR,^194^ adding an additional layer of complexity to the role and mechanism of WNT pathway in hepatic function and metabolism.

The WNT pathways also modulate adipose IR^185^ and adipogenesis,^195^ notwithstanding the diverse regulatory roles of different WNT pathways. It has been disclosed that the activation of WNT5a/PCP pathway could promote adipose IR via inducing adipose inflammation.^196^ Concomitant with this, sFRP5, a protein counteracting WNT5a/PCP activation, is able to suppress inflammation by blocking the JNK pathway, and thus improve glucose and insulin intoleration in obese mice.^197^ Additionally, WNT pathways in the brown adipose tissue (BAT) also regulate IR progression. For instance, knockdown of LRP6, a receptor of WNTs, was shown to improve BAT insulin sensitivity through increasing the expression of PGC1α and uncoupling protein 1 (UCP1).^198^ In terms of adipogenesis, it appears that different WNT members have distinct functional outcomes. Basically, several WNT members, including WNT3a, WNT6, WNT8, WNT10a, and WNT10b, have been observed to suppress adipogenesis in the β‐catenin‐ or PCP‐dependent ways,^184^, ^199^ while others, such as WNT4, WNT5a, WNT5b and WNT11, are capable of stimulating adipogenesis.^195^, ^200^, ^201^ Given the complexity of WNT members, it is of interest to dissect their real roles in adipogenesis under diverse physiological and pathological conditions.

The WNT10b/β‐catenin pathway is depressed in the skeletal muscle tissues of overweight and prediabetes. Therefore, not surprisingly, activating WNT10b/β‐catenin pathway could improve insulin sensitivity in the skeletal muscle, which is dependent on a reduction in the lipid deposition of myoblasts, which is regulated by SREBP1c.^202^ Similarly, activation of WNT/PCP pathway is also related to an improved insulin sensitivity of skeletal muscle.^193^ However, another WNT antagonist, secreted frizzled‐related protein 3 (sFRP3), was significantly reduced in the skeletal muscle of prediabetes and T2DM, leading to impaired insulin sensitivity in T2DM,^203^ recapitulating the distinct roles of different WNT members in adipogenesis. In summary, the functions of WNT pathways in skeletal muscle IR may vary with different pathways, as well as with different contexts.

The WNT pathways are also important for β cell fate and insulin secretion.^204^ Several WNT pathways, such as WNT3a/β‐catenin^204^, ^205^, ^206^ and WNT4/PCP,^207^ could improve β cell proliferation and insulin secretion. In‐depth dissections revealed that the activation of TCF7L2 and FoxO1 is key to this proproliferation effect of WNT3a/β‐catenin pathway.^187^, ^208^, ^209^ TCF7L2 is crucial to maintaining the normal functions of β cells, and its silencing can inhibit GSIS through regulating the expression of genes that control the fusion of secretory granule, such as syntaxin 1A, and syntaxin‐binding protein 1.^210^ As to the WNT4/PCP pathway, its promotive effect on β cell proliferation relies on JNK activation and the increment of NK6 homeobox 1 and PDX1 protein.^207^ In addition, the WNT/Ca^2+^ pathway may contribute to insulin secretion, as inactivation of Ca^2+^ and NAFT could diminish the biosynthesis of dense core granule.^211^ Nonetheless, some WNTs ligands might exert counteracting effects on β cell proliferation and function, raising the possibility that the uncontrolled imbalance of WNTs might be responsible for the deficiency of functional β cells during T2DM progression.^185^, ^212^

### 4.5 UPR pathway

**FIGURE 8:**
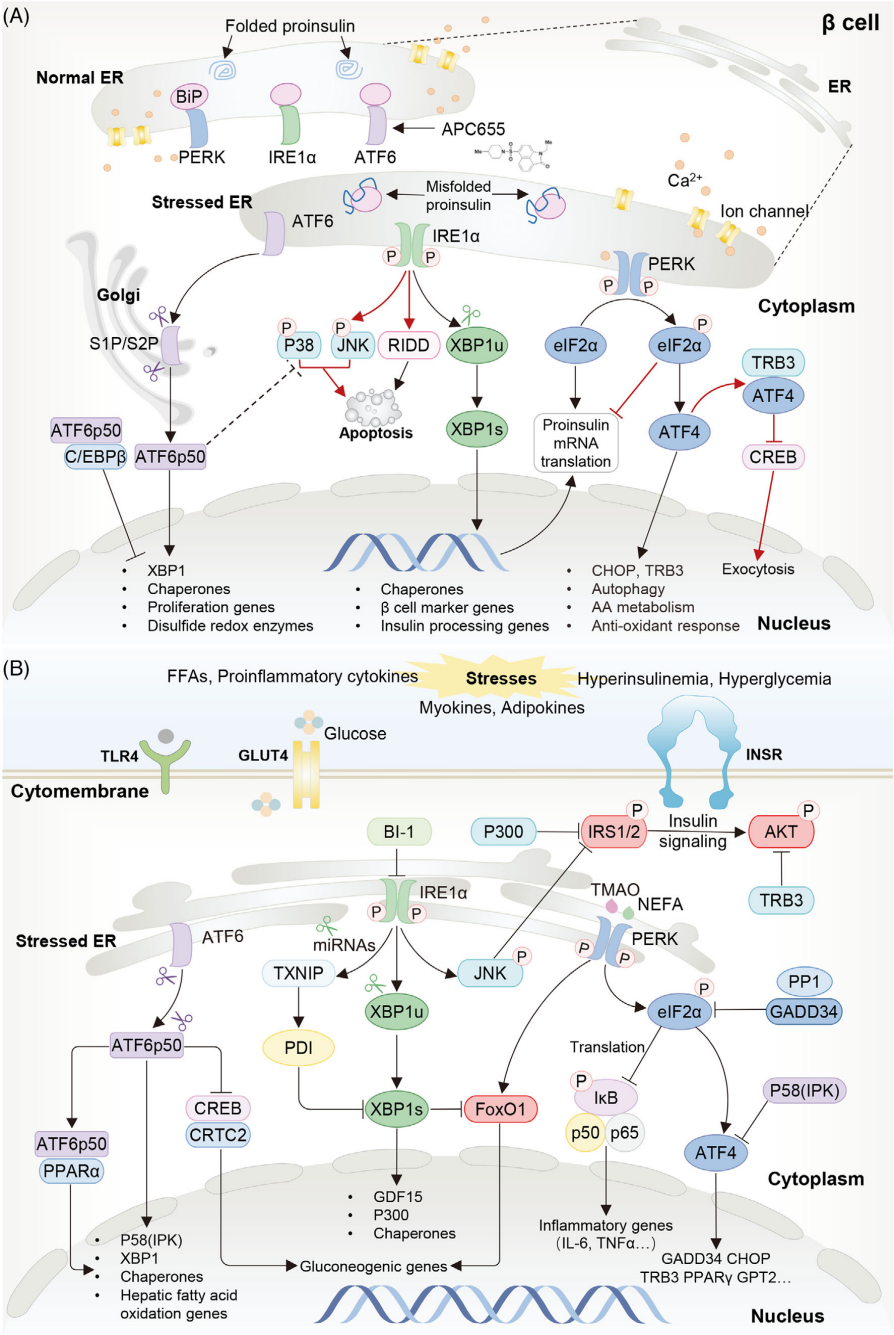
The UPR pathway in T2DM. (A) Beneficial and harmful effects of UPR activation in regulating β cell function and survival. Adaptive UPR plays a role in β cell survival, proliferation, and identity by promoting the transcription of related genes through ATF4/6 and XBP1s, and by suppressing global translation. Black lines indicate beneficial effects of UPR. Chronical or terminal UPR leads to β cell dysfunction and failure. Chronical PERK activation results in translational suppression of required genes, including chaperones, insulin, and ER enzymes, as well as induction of cell death through CHOP. Besides, ATF4 can interact with its target TRB3 to inhibit the transcriptional activity of CREB, leading to the reduction of exocytotic gene expression. Hyperactivation of IRE1α leads to RIDD process, which cleaves numerous mRNAs including genes related to protein folding, β cell maturation and cell survival, and the activation of JNK and p38. All these finally lead to excess β cell death. Red lines indicate harmful effects of UPR. (B) Model recapitulating the interplay between UPR and insulin response in peripheral tissue. During T2DM, elevated circulating factors, such as glucose, cytokines, and FFAs, can trigger ER stress, which activates the three UPR branches. Activated PERK/eIF2α/ATF4 arm affects insulin sensitivity. On the one hand, the UPR may modulate the transcription of genes associated with inflammation, ER stress and insulin response, such as TRB3, an endogenous inhibitor of AKT. On the other hand, activated PERK can directly regulate the expression and activity of FoxO1, thereby enhancing insulin resistance. IRE1/XBP1 impairs insulin signaling through enhancing the activation of JNK and transcription of P300. Both JNK and P300 blunt the activation of IRS1/2. Inhibition of IRE1α activity by BI‐1 increases hepatic insulin sensitivity and glucose homeostasis. ATF6 branch protects organs from insulin resistance by inhibiting CREB activity, increasing PPARα transcriptional capacity and the expression of chaperones and PERK arm inhibitor P58(IPK).

The UPR pathway can be activated by the perturbation of ER homeostasis, which is characterized by the accumulation of unfolded/misfolded proteins, to alleviate the stress of the ER or cause cell death. The UPR cascade encompasses three upstream branches to sense the ER transmembrane stress: inositol‐requiring enzyme 1 (IRE1), protein kinase R‐like ER kinase (PERK), and activating transcription factor 6 (ATF6) (Figure 8A).^213^, ^214^ Activated IRE1α could produce a transcriptionally active XBP1s,^215^, ^216^ which further overcomes the ER turbulence by inducing the transcription of genes associated with protein folding, translocating, trafficking, and ER‐associated degradation,^217^ as well as by interacting with several signaling pathways, such as p38/MAPK and PI3K pathways.^213^ If ER stress is not mitigated, IRE1α would become hyperactivated and oligomerized to degrade hundreds of ER‐localized mRNAs for relieving the folding burden on ER,^213^, ^214^ or induce cell death^213^, ^214^ by degrading certain miRNAs targeting proapoptotic genes and activating apoptosis signal‐regulating kinases.^218^ Activated PERK could phosphorylate eukaryotic translation initiation factor 2 (eIF2α) to attenuate global protein translation.^213^, ^214^ By selectively upregulating the expression of activating transcription factor 4 (ATF4), the PERK pathway enhances the transcription of growth arrest and CHOP/GADD34 to negatively regulate itself and induce cell death respectively.^214^, ^219^ During ER stress, ATF6 can be transported to the Golgi apparatus and then be cleaved to release the transcriptionally active ATF6(p50) cytosolic fragment.^220^ The active fragment is translocated to nucleus and then regulates the transcription of multiple genes involved in increasing ER protein‐folding capacity, including XBP1s, to relieve ER stress.^221^ To date, the importance of ER stress in T2DM progression^37^ has inspired a consensus that activation of UPR pathway is an emblematic phenomenon in T2DM‐associated dysmetabolic outcomes, which in turn controls peripheral IR and β cell dysfunction via various mechanisms.

Most UPR components, induced by increased ER stress, have been observed to be upregulated in peripheral tissues during obesity and T2DM (Figure 8B).^39^, ^222^, ^223^ Subsequently, the UPR pathway is able to modulate IR in the liver,^224^ adipose tissue, and skeletal muscle,^224^ as well as adipogenesis,^225^ despite certain inconsistent roles among different branches. Activated PERK pathway probably increases hepatic IR, as evidenced by that liver‐specific depression of the PERK/eIF2α/ATF4 pathway by GADD34 overexpression or ATF4 depletion could improve IR and glucose intolerance in diet‐induced obese (DIO) mice.^226^, ^227^ The underlying mechanisms might be associated with transcriptional regulation of numerous targets, such as tribbles homolog 3 (TRB3) (an endogenous inhibitor of AKT),^228^, ^229^ PPARγ,^230^ and glutamic pyruvate transaminase 2 (GPT2) (a promotor of gluconeogenesis).^231^ In addition, PERK may counteract the effect of AKT by potentiating FoxO1 activity.^232^ Notably, PERK also acts as a receptor for the gut‐microbe‐derived metabolite, trimethylamine N‐oxide,^233^ which is increased in IR and associated with several complications of metabolic syndrome in human.^234^ In contrast, it has also shown that activation of the eIF2α/ATF4 pathway by the heme‐regulated eIF2α kinase (HRI) can promote the expression of FGF21, a metabolism‐beneficial liver hormone, thereby reducing glucose intolerance in DIO mice,^235^ and ensuring the effects of metformin on appetite and weight loss,^236^, ^237^, ^238^ indicative of the ambiguous roles of PERK pathway in T2DM.

The IRE1α branch is also abnormally activated in the liver with IR/hyperinsulinemia, along with increased XBP1s splicing and nuclear localization.^239^ Intriguingly, activation of IRE1α by insulin plays protective effects on hepatic insulin action and glucose homeostasis,^240^ which may be related to activating growth differentiation factor 15 (GDF15) transcription,^241^ driving hepatic autophagy,^242^ relieving ER stress, and/or decreasing FoxO1 expression.^243^ However, IRE1α/XBP1s activation was also found to blunt insulin signaling and exacerbate IR in the liver^244^ by reducing expression of the proinflammation factor, Bax inhibitor 1 (BI‐1),^245^ and inducing the acetylation of IRS1/2.^246^

Distinct to the PERK and IRE1α branches, the ATF6 is decreased in the liver of diabetic mice,^247^ and potentially functions protectively in hepatic glucose metabolism. Indeed, hepatocyte‐specific overexpression of ATF6α reduced hepatic glucose output and steatosis,^248^ while its whole‐body deletion exacerbated glucose intolerance.^249^ Mechanistically, ATF6 can suppress the transcription of gluconeogenic genes by disrupting the interaction between CREB and CREB‐regulated transcription coactivator 2 (CRTC2),^250^ promote hepatic FA oxidation by interacting with PPARα,^248^ and suppress the PERK/eIF2α/ATF4 branch by promoting P58(IPK) expression.^228^

In obese individuals, chronic ER stress and UPR pathways in the adipose tissue can be sustainedly activated by increased circulating levels of FFAs^251^ and insulin,^252^ which in turn impairs insulin signaling of adipose tissue. For example, the IRE1α/JNK1 axis inactivates IRS1,^253^ and the PERK/ATF4/TRB3 axis induces AKT suppression,^222^ abolishing insulin sensitivity and glucose transport in the adipose tissue. Adipose UPR pathways also contribute to insulin desensitization in other organs, for one reason that ER stress is linked to reduced adiponectin secretion,^254^, ^255^, ^256^ and for another reason that the PERK arm can elevate circulating levels of tumor necrosis factor α (TNFα), interleukin‐6 (IL‐6), and IL‐1β.^251^, ^257^ All of these impose a vicious ER stress feedback and further exacerbate the adipose tissue per se and systemic IR.^258^

In the skeletal muscle, aberrant activation of UPR pathways is also found in patients with T2DM and pregnant women with obesity or gestational diabetes.^223^, ^259^ These abnormalities impair muscular insulin action via the mechanisms that resemble those in the adipose tissue.^259^ That is, both PERK‐induced TRB3 activation^259^ and IRE1α/JNK‐triggered IRS1 phosphorylation can contribute to muscular IR.^40^ Furthermore, transcriptionally activated ATF6/XBP1s pathway warrants increased expression of skeletal muscle kidney‐enriched inositol polyphosphate phosphatase (SKIP) to induce muscular IR.^260^ Of note, activation of IRE1α/JNK axis also bridges the increased release of IL‐6 and TNFα from skeletal muscle and the disrupted systemic insulin sensitivity in T2DM.^261^, ^262^ In addition, activated PERK is implicated in the secretion of myokines, including musclin and ceramides,^263^, ^264^ which are key to insulin desensitization in peripheral organs.^265^ In contrast, inhibition of ER stress significantly enhances the expression of UCP1 in the inguinal WAT and improves metabolic phenotypes in DIO mice,^266^ which is linked into a decrease in the JNK‐mediated degradation of PPARγ. Moreover, the ATF6 branch might regulate multiple processes in the skeletal muscle, including exercise training adaption,^267^ glucosamine‐induced disruption of glucose uptake^268^ and apoptosis,^269^ all of which are closely related to T2DM.

On the road to T2DM, proinsulin is prone to misfolding, and excess insulin production can aggravate ER stress in β cells.^37^ In parallel, dysregulations of UPR pathways, for example, transcriptional dysregulation related to pancreatic aging,^270^ local inflammation^271^ and glucotoxicity,^37^ are also observed in the islets of T2DM patients and rodents,^272^ which possibly exert effects on β cell dysfunction by regulating insulin production and cell fate. Supporting this notion, the PERK pathway is considered to act as a metabolic sensor to modulate insulin production and secretion in a delicate way. In detail, although ablating PERK is shown to impair insulin trafficking and β cell survival, leading to insulin insufficiency and hyperglycemia,^273^ partial attenuation of PERK activity instead enhances GSIS through regulating ER chaperones and Ca^2+^ transit.^274^, ^275^ Moreover, both β cell‐specific ablating^276^ and enhancing phosphorylation^277^ of eIF2α cause reduced insulin secretion, increased β cell apoptosis and thus severe diabetes. Besides, PERK/ATF4/TRB3 axis acts through inducting CREB inhibition to depress the transcription of key exocytosis genes, and consequently reduce insulin secretion.^278^ Meanwhile, the IRE1α pathway also appears to ensure β cell function. The IRE1α/XBP1s axis promotes ER protein folding capacity by regulating the transcription of genes involved in insulin folding, process and degradation,^279^, ^280^ and thus improves insulin production and secretion.^281^, ^282^ Supporting this, XBP1s deficiency in β cells markedly blunts GSIS,^280^ increases β cell apoptosis by deactivating β cell identity genes,^283^ and enhances inflammation and oxidative stress.^281^ Conversely, prolonged XBP1s production in rat β cells can inhibit the expression of β cell markers, and eventually lead to β cell apoptosis.^284^, ^285^ The ATF6 pathway has an essential role in supporting β cell function and survival by inducing the transcription of target genes, including ER chaperones,^275^ disulfide redox enzymes, and several quality control and degradation factors, as well as XBP1s.^37^ In line with this, whole‐body deletion of ATF6 impaired glucose intolerance and blunted insulin secretion,^249^ whereas ATF6 induction improved β cell insulin secretion and viability under ER stress conditions.^286^ Furthermore, inhibiting ATF6 could result in embryonic lethality and β cell receding by arresting cell cycle entry,^287^ while upregulating ATF6α activity is able to markedly expand β cell mass in db/db mice,^288^ suggesting its importance in the fate control of β cells.^289^, ^290^ Of note, ATF6 also participates in β cell proliferation induced by the salt‐inducible kinases inhibitor, known as HG‐9‐91‐01, and knockdown ATF6 can efficiently reverse such proproliferating effect.^291^

### 4.6 Hippo pathway

The Hippo signaling pathway, mainly comprising macrophage stimulating 1/2 (MST1/2), large tumor suppressor 1/2 (LATS1/2), and their cofactors such as salvador homolog (SAV) and MOB kinase activator 1A/B (Mob1A/B), predominantly controls the activity of YAP/PDZ‐binding motif (TAZ), two closely related mammalian transcriptional coactivators that shuttle between the cytoplasm and nucleus (Figure 7B).^292^, ^293^, ^294^ Generally, active MST1/2 phosphorylates LATS1/2, which in turn phosphorylates YAP/TAZ and thus inhibits the activity of YAP/TAZ.^292^ In contrast, dephosphorylation allows YAP/TAZ to translocate into the nucleus and combine with its partner transcriptional enhanced association domain (TEAD) to reprogram gene expression.^292^ However, the upstream regulation of YAP/TAZ is beyond the Hippo pathway, especially when cells are stimulated by those uncanonical signals such as mechanical stimuli, G protein‐coupled receptor (GPCR) ligands, metabolites, and cell stresses.^292^, ^293^, ^294^ Currently, deciphered regulatory loops that involve many kinds of metabolic responses gradually related YAP/TAZ and Hippo pathway to the peripheral glucose metabolism, insulin signaling, the fate of β cells, and thus pathological processes of T2DM.^293^, ^294^, ^295^

Cells may employ the YAP/TAZ as a coordinator to balance glucose energy supply and consumption.^295^ Glucose deprivation, reduced glucose uptake and inhibited glycolysis could repress the activity of YAP/TAZ, the formation of the YAP/TEAD complex, and thus suppress the transcription of target genes,^296^, ^297^, ^298^ such as GLUT3, hexokinase 2 (HK2) and phosphofructokinase B3 (PFKB3), as well as to diminish glucose consumption. Mechanistically, deficient glucose metabolism could act through the activation of AMPK and LAST1/2 to inhibit YAP/TAZ activity, and could also depress the phosphofructokinase 1 (PFK1) to prevent the formation of the YAP/TEAD complex.^296^ In contrast, high glucose could upregulate HBP‐dependent O‐GlcNAcylation of YAP, which enhances YAP activity by restraining LATS‐dependent phosphorylation and proteasomal degradation.^299^ Given that blood glucose level after long‐standing fasting and postprandial during T2DM is associated with dysregulated cellular glucose metabolism, it is possible that the Hippo pathway may modulate T2DM progression via regulating cellular glucose metabolism.

YAP is decreased in the skeletal muscle of obese patients and mice with IR.^300^ Consistent with this, the YAP/TAZ pathway might promote peripheral insulin signaling and repress glucogenesis, whereas the Hippo pathway works inversely. For example, YAP/TAZ might upregulate the insulin/AKT pathway by potentiating IRS2 transcription in the liver of mice with codeleted PTEN and SAV1.^301^ Moreover, the mutually concordant positive effects between YAP/TAZ and mTOR pathways also underpin the connection between YAP/TAZ and the insulin/AKT signaling.^302^ On the contrary, the Hippo pathway exerts converse effects on insulin signaling and glucose metabolism. One evidence is that upregulating the upstream Hippo kinase MST3 could exacerbate IR, hyperglycemia and hyperinsulinemia by depressing IRS1 and upregulating the transcription of gluconeogenic regulators and enzymes.^303^ YAP can also act through PGC1α to suppress the transcription of hepatic gluconeogenic genes, lower plasma glucose level and improve glucose tolerance.^304^ Additionally, glucagon activates LATS and restrains YAP,^305^ which in return eradicates upregulation of hepatic gluconeogenic genes and represses gluconeogenesis.^304^

The Hippo pathway may also serve as an essential regulator in the capacity of adipose tissue to endure metabolic stress.^306^ To deal with the increasing stress during obesity, WAT might rely on YAP/TAZ to resist apoptosis and ameliorate T2DM.^38^, ^306^ Additionally, it has shown that the impairments of FA oxidation and consequent enhanced adiposity of skeletal muscle in obese patients or prediabetic mice are caused partially by reduced YAP.^300^ Intriguingly, the Hippo pathway may also participate in inflammatory responses^300^, ^307^ and mitochondrial maintenance, bringing about an attractive notion that dysregulation of YAP/TAZ and Hippo pathway might influence the advancement of T2DM in the manners beyond our imagination.

The Hippo pathway and YAP/TAZ also modulate pancreatic cell differentiation, proliferation and apoptosis.^293^, ^308^ Overall, YAP/TAZ could render expansion of early embryonic pancreas epithelium, but hamper endocrinogenesis of pancreatic progenitor cells.^308^ In harmony with this, YAP/TAZ in adult pancreatic β cells remains low expression level,^308^ indicating that silencing of YAP/TAZ is critical for the maturation of β cells. In contrast, the Hippo pathway^309^ tends to induce apoptosis of β cells, as MST1 is activated to empower β cell apoptosis, while its deletion pronouncedly restores β cell function and mass, attenuating diabetic conditions in mice with T2DM.

### 4.7 HIFs pathway

HIFs are a family of DNA binding transcription factors activated by hypoxia in mammalian, among which HIF1α and HIF2α are best‐studied and have been reported to play critical roles in several diseases, such as T2DM, atherosclerosis and cancer.^310^ Under hypoxic conditions, HIF1α maintains stability via limited oxygen and could be translocated to the nucleus to bind to HIF1β and other response elements,^311^ thus augmenting the activation of its target genes transcriptionally, such as vascular endothelial growth factor (VEGF), angiopoietin, and platelet‐derived growth factor.

Previous evidence demonstrated that the impaired HIFs signaling pathway acts as one of the key pathogenic factors of T2DM, and is involved in IR.^310^, ^312^ In hepatocytes, HIF1α modulates glucose transport and fructose production by regulating its downstream targets, such as GLUT1 and PDK1.^310^ HIF2α could regulate gluconeogenesis and HGP through the IRS2/PI3K/AKT pathway.^313^ Activated HIF2α by refeeding is also able to attenuate postprandial glucagon signaling through upregulating cAMP level and inhibiting CREB activity, ultimately repressing the expression of PEPCK and G6Pase.^314^ Meanwhile, intestine HIF2α could inhibit the expression of neuraminidase 3 (Neu3), thus substantially ameliorating hepatic steatosis, glucose intolerance and IR.^315^ Adipocyte‐specific knockout HIF1α and HIF1β in HFD‐fed mice can enhance glucose tolerance and insulin sensitivity by inducing tyrosine phosphorylation of signal transducer and activator of transcription 3 (STAT3) and suppressor of cytokine signaling 3 (SOCS3)‐mediated increase of adiponectin.^316^ Consistently, decreased insulin sensitivity and glucose intolerance could be observed in mice with WAT‐specific HIF1α overexpression.^317^ Notably, as essential mediators of adaptation to hypoxia, HIFs pathways also play a key role in controlling mitochondrial functions and ROS production, which potentially complicate the interactions between mitochondrial dysfunction and glucose metabolism and T2DM.^318^

Disruption of HIFs homeostasis affects insulin secretion.^319^ HIF1β is significantly down‐regulated in islets of T2DM.^320^ Specific knockout of HIF1α in β cells impaired insulin secretion and decreased glucose‐stimulated ATP production.^319^ However, overload of HIFs could also lead to β cell dysfunction. For example, deletion of von Hippel–Lindau factor, a regulator of HIF hydrolysis, markedly impaired insulin secretion and glucose homeostasis in mice, accompanied with increased HIFs levels.^321^ These results suggest that proper levels of HIFs might be critical to maintaining β cell homeostasis and function, which awaits further investigation.

### 4.8 TGFβ pathway

The TGFβ superfamily contains a number of subfamily proteins, such as bone morphogenetic proteins (BMPs), activins and TGFβs (Figure 7D). Activation of TGFβ receptors could further activate small mothers against decapentaplegic homolog (SMADs), a class of second messengers, and other signaling pathways, such as MAPK, RHO GTPase, and PI3K/AKT pathways.^322^ The TGFβ pathway connects contextual determinants with specific cellular responses by controlling SMAD‐dependent transcription programming and integrating with other pathways. These fundamental mechanisms underlie the function of TGFβ pathway in controlling peripheral insulin signaling and pancreatic β cell biology during T2DM pathogenesis.

An increasing body of evidence supports that the TGFβ pathway can affect glucose homeostasis. It is increasingly clear that several members of the TGFβ superfamily, including activin A and B, GDF11, BMP2‐4, and TGFβ1‐3, have emerged as novel regulators in the insulin signaling of adipose tissue, skeletal muscle, and liver.^323^ Among which, the bioactivity of the activin/GDF11 is reported to be modulated by the antagonists follistatin (FST) and follistatin like 3 (FSTL3), which could increase fat mass and adipose IR, and therefore disrupt glucose homeostasis.^324^ Specifically, overexpression of *fstl3* in obese mice is capable of improving muscle insulin sensitivity and reducing fat accumulation, but enhancing hepatic glucagon sensitivity.^325^ By contrast, genetic removal of *fst* enhanced WAT insulin sensitivity and suppressed HGP, thereby ameliorating glucose tolerance in obese mice.^326^ BMPs, particularly BMP4, 6, and 7, are also involved in the control of glucose homeostasis. Specifically, BMP7 promotes, while BMP4 decreases insulin sensitivity in the adipose and muscle of T2DM mice, respectively.^327^ Mechanistically, BMP7 can activate PDK1 and AKT to boost GLUT4 translocation to the plasma membrane and in turn increase glucose uptake, while BMP4 counts on the activation of PKCθ to induce IR. BMP6 may restore the levels of blood glucose and lipids in T2DM mice via reducing hepatic gluconeogenesis and glucose output.^328^ However, the dysregulation of TGFβ pathway in the diabetic peripheral tissues remains incompletely understood and awaits further study.

The discoveries about the roles of TGFβ in pancreatic development and functions establish another strong link between the TGFβ pathway and T2DM,^329^ regardless of the diversity of TGFβ pathway in controlling the proliferation, apoptosis and differentiation of β cells. To be specific, activin A could inhibit β cell proliferation, but facilitate the generation of β cells from adult stem cells, human embryonic stem cells, ductal cells, human amniotic epithelial cells),^330^ and α cells.^331^ Activin A and activin B potentiate the dedifferentiation of β cells by depressing and upregulating the transcription of crucial genes associated with β cell maturity and immaturity, respectively.^332^ Activation of SMADs is key to the regulation of TGFβ signaling on β cell fate. In detail, SMAD7 is believed to enhance proliferation,^333^ while SMAD2 and SMAD3 block proliferation of β cells by regulating the nuclear localization of p27, a cell‐cycle modulator inhibiting cyclin‐dependent kinase (CDK). Otherwise, activation of TGFβ/SMAD3 signaling also has a role in β cell apoptosis.^334^ Furthermore, SMAD2, 3, and 7, particularly SAMD7, connect the dedifferentiation and proliferation of β cells together.^335^ Recently, the roles of TGFβ pathway in controlling β cell functions are also emerging. For instance, activin A/B, TGFβ1, FST, GDF11, and BMPs, have been demonstrated to enhance GSIS,^336^, ^337^, ^338^ while the addition of FSTL3 is able to reverse these effects in both functional and nonfunctional islets.^338^ Mechanistically, these effects may be predominantly attributed to the activation of SMAD2, since its disruption could compromise insulin secretion under high glucose and diabetic conditions.^337^ Furthermore, the effects of TGFβ pathway on GSIS may be associated with the modulation of K_ATP_ and calcium channels activity,^337^ as well as the altered expression of essential genes governing insulin secretion, such as the GLUT2 and calcium voltage‐gated channel subunit alpha1 D genes.^338^

### 4.9 FGFs pathway

**FIGURE 9:**
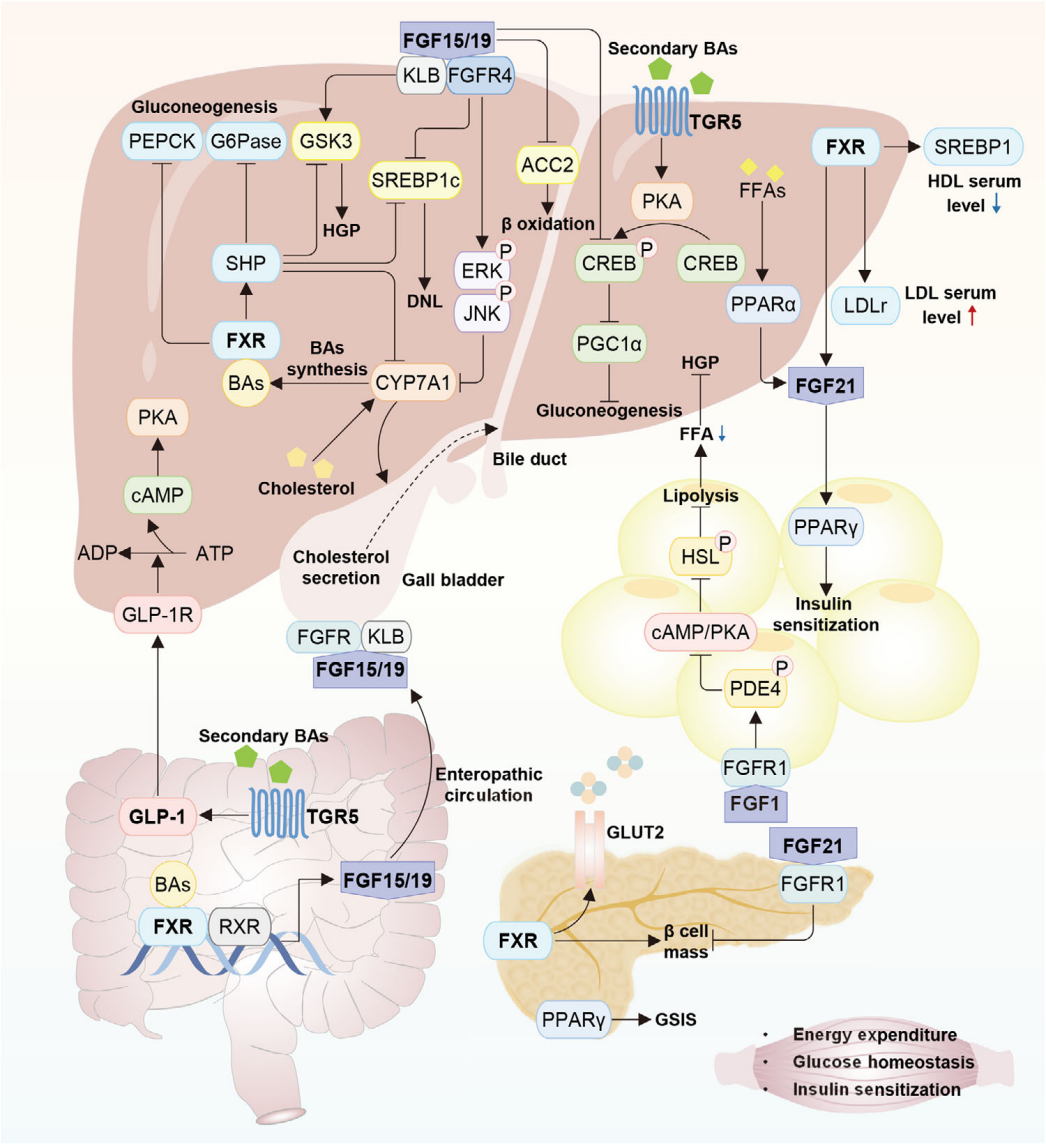
Crosstalk among bile acids, FGFs, and GLP‐1 pathways in T2DM. Several important signaling pathways are interacted in multiple metabolic tissues. In hepatocytes, FXR induces SHP, subsequently inhibits CYP7A1 transcription and BA synthesis. Under BAs stimuli, FXR inhibits the transcription of PEPCK and G6Pase to suppress gluconeogenesis, represses GSK3 to suppress hepatic glycogen production (HGP), and suppresses SREBP1C to regulate de novo lipid synthesis (DNL). In the ileum, BAs are reabsorbed into enterocytes and then enter circulation induced by intestinal FXR. Together, activated FXR could reduce serum glucose level. In addition, FGF15/19 is secreted to the blood and then circulates to the liver, where FGF15/19 binds to its receptors, subsequently inhibiting BA synthesis and meanwhile regulating downstream molecules in metabolic tissues, including ACC1, GSK3, CREB, and SREBP1c. However, FGF21 stimulates glucose uptake in the adipose tissue, but inhibits β‑cell proliferation in pancreatic islets. Interestingly, activated TGR5 promotes the secretion of FGF19 and GLP‐1 to blood and mediates downstream functions. PPARs have been characterized as core lipid sensors that modulate whole‐body energy metabolism. Collectively, the cooperation among BAs receptors, FGFs, GLP‐1, and PPARs maintains the glycolipid metabolic responses in T2DM. The online resource inside this figure was quoted or modified from Scienceslide2016 plug‐in.

The human FGF superfamily consists of 22 structurally related signaling molecules.^339^ FGFs exert their pleiotropic effects by dimerizing, activating and phosphorylating FGF receptors, leading to the activation of the RAS/MAPK, PI3K/AKT, Ca^2+^, PKC, and STAT signaling cascades in a cellular context‐dependent manner (Figure 9).^340^ Most FGFs family members canonically present in the extracellular matrix (ECM) and act in autocrine and/or paracrine manners to activate FGFRs.^341^ While FGF19 subfamily members, including FGF19 (the mouse ortholog FGF15), FGF21, and FGF23, are liberated from the ECM into the bloodstream and thus work in an endocrine manner.^342^ Tremendous efforts throughout the past decades have ascertained the pleiotropic effects of FGFs pathways on T2DM progression, potentiating the development of engineered FGF analogs and mimetics targeting T2DM.^343^, ^344^, ^345^

Several subclasses of FGFs pathways have been disclosed to impose beneficial impacts on peripheral insulin action and glucose homeostasis. Among them, FGF21 has obtained the most interests owing to its powerful effects on favoring insulin sensitivity and glucose metabolism within the liver and adipose tissue.^343^, ^346^ As a stress‐inducible hormone, FGF21 is strongly induced by starvation, amino acid restriction, ketogenic diet and HFD treatment in the liver. In the liver, by binding to FGF receptor 1c (FGFR1c) and the coreceptor β‐Klotho,^347^ FGF21 can elicit the FGF signaling to increase insulin sensitivity by suppressing mTORC1,^348^ reduce lipid accumulation by promoting PGC1α‐induced FA oxidation^349^ and repressing SREBPB1c‐mediated lipogenesis,^350^ and block TG‐enriched very‐low‐density lipoprotein (VLDL) uptake by decreasing VLDL receptor expression.^351^ In the adipose tissue, FGF21 may stimulate glucose uptake in an insulin‐independent manner but instead through inducing GLUT1 expression, inhibit lipolysis by sequential activation of ERK1/2 and serum response factor/Ets‐like protein‐1 (SRF/Elk‐1),^352^, ^353^, ^354^ and promote adiponectin production and secretion from adipocytes through a PPARγ‐dependent mechanism.^355^, ^356^ Paradoxically, the endogenous expression and circulating level of FGF21 are increased in obese humans and mice, which is likely linked to the chronic mild mitochondrial dysfunction.^357^

The FGF15/19 signaling is another important pathway for peripheral insulin action and glucose metabolism. After feeding, FGF15/19 is released postprandially from the small intestine to serum and arrived at other organs.^358^ In the liver, FGF15/19 binds to its coreceptors, FGFR4 and β‐Klotho, to trigger ERK pathway and consequently phosphorylate and inactivate GSK3 in an insulin‐independent manner, ultimately increasing the hepatic GS activity.^359^ Meanwhile, FGF15/19 can suppress gluconeogenesis through a mechanism involving the inactivation of CREB and subsequent downregulation of PGC1α.^358^ Additionally, FGF15/19 is strongly induced by the nuclear receptor farnesoid X receptor (FXR) in the small intestine to repress hepatic BAs synthesis.^360^ Beyond these, FGF15/19 also regulates postprandial glucose and energy homeostasis^361^ through modulating the synthesis of proteins, glycogen and glucose.

In addition to aforementioned FGFs, FGF1 is emerged as a potent regulator in peripheral glucose homeostasis.^344^ Both acute and chronic treatment with recombinant FGF1 can normalize blood glucose concentration, at least partially attributed to suppressing HGP and promoting insulin‐dependent glucose uptake in skeletal muscle.^362^ At the same time, FGF1 may activate the phosphodiesterase 4D (PED4D) and then repress cAMP/PKA axis to inhibit lipolysis in adipose tissue and suppress HGP.^363^ Of note, FGF1 expression in adipose tissue has been reported to be controlled by PPARγ,^364^, ^365^ suggesting that the PPARγ/FGF1 axis might be another crucial mechanism for systemic insulin sensitivity. Similar to FGF1, FGF4 may also function as a potent anti‐hyperglycemic factor, and paracrine FGF4 exhibits higher hypoglycemic capacity even than endocrine FGF21.^366^ Correspondingly, FGF4 treatment is able to effectively improve IR in genetic‐induced obese mice through many potential mechanisms, for example, activating AMPK in the skeletal muscle to promote GLUT4 expression and its translocation to cell membrane.^366^

The FGF pathway also confers metabolic benefits in β cells function and survival, as well as central neural regulation.^344^ For instance, FGF21 knockout mice display islet hyperplasia and distort morphology, along with increased β cell proliferation and a larger α‐cell population, while islets and cultured β cells treated with FGF21 are partially protected from apoptosis possibly due to the activation of PI3K/AKT signaling.^367^ In addition, FGF15/19 could reduce food intake and improve glucose tolerance by inhibiting the hypothalamic–pituitary–adrenal axis.^368^, ^369^, ^370^ Moreover, glucose can induce the release of FGF1 into the cerebrospinal fluid, which further initiates feeding suppression and glycemic control by interrelating with tanycytes, astrocytes and glucose‐sensing neurons of the hypothalamus.^344^

### 4.10 Bile acids

BAs, the major components of bile, are synthesized from cholesterol in liver via the classical pathway regulated by cholesterol‐7α‐hydroxylase (CYP7A1).^371^ BAs play important roles in facilitating the absorption of dietary lipids, maintaining cholesterol homeostasis and activating hormones (Figure 9). Over the past decades, BAs have been regarded as critical regulators in glucose, lipid and energy metabolism, as well as potential targets for preventing cardiovascular complications and improving glycemic control in T2DM patients.^372^

BAs act primarily through activating intracellular ligand‐activated receptors, among which the best‐studied are FXR and G protein‐coupled BA receptor (TGR5). FXR (also known as NR1H4) is a member of nuclear receptor family and expressed in many tissues.^373^ FXR located in the intestine and liver, could be activated directly by BAs and forms a heterodimer with retinoid‐X‐receptor (RXR). FXR exerts effects on the expression of downstream genes and the inhibition of BAs synthesis through two ways: (1) inducing the small heterodimer partner (SHP) to inhibit CYP7A1 in the liver; (2) increasing circulating FGF19/15 in the gut to repress CYP7A1. TGR5, belonging to the rhodopsin‐like superfamily of G protein‐coupled receptors, is a cell membrane receptor for secondary BAs and moderately expressed in nearly all tissues. BAs could regulate glucolipid metabolism via binding to FXR and TGR5.^374^ For example, activated FXR is able to repress hepatic DNL^375^ and gluconeogenesis^376^ by inhibiting the expression of SREBP1c, PEPCK, and G6Pase. Moreover, systematic knockout of TGR5 could aggravate IR and glucose intolerance through miR‐26a and the subsequent cAMP/PKA pathway.^377^, ^378^ Furthermore, both FXR and TGR5 are capable to regulate GSIS in pancreatic β cells through inducing the relocalization of GLUT2 on the membrane.^379^

TGR5 is coexpressed with FXR on L cells in the intestine, and the activation of FXR could facilitate the release of GLP‐1,^380^ which then enhances insulin secretion and improves glucose homeostasis. Intriguingly, hyocholic acid has been shown to promote GLP‐1 secretion through synchronously activating TGR5 and inhibiting FXR, which exerts profound effects on glucose metabolism in the pig and diabetic mouse models.^381^ As well, a recent study showed that soluble dietary fiber oligofructose could activate TGR5 to enhance GLP‐1 activity by stimulating 6α‐hydroxylated BAs, thus improving host glucose metabolism and maintaining glucose homeostasis.^382^ All of above suggest that BAs signaling pathways mediated by intestinal GLP‐1 have strong potential for T2DM therapeutic applications.

The gut microbiota has been showed to regulate the level and composition of BA metabolites through secreting enzymes to catalyze the dehydroxylation of BAs, thus influencing the function of BAs in regulating glycolipid metabolism. In turn, BAs also interact with and affect the gut microbiota,^383^ thus creating a dynamic equilibrium between them. Interestingly, metformin has been reported to decrease the abundance of species of B. fragilis in the intestine to upregulate the bile acid glycoursodeoxycholic acid in the gut, thus improving metabolic dysfunction and hyperglycemia in T2DM patients.^384^ Adjustment of BAs homeostasis has been applied to optimize glycaemia parameters and improve pathological conditions in various diabetic animal models and human patients,^385^, ^386^ which implies potential therapeutic roles for BAs signaling in the treatment of T2DM.

### 4.11 Ca^2+^ signals

Ca^2+^ signals are finely tuned by a huge group of proteins, including channels, pumps, transporters, and binding proteins,^387^, ^388^ which underpin the accurate influx and reflux of Ca^2+^.^387^ Ca^2+^ homeostasis is key to the well‐proceeding of many cellular processes,^387^, ^388^ including insulin secretion, β cell mass, insulin sensitivity, and glucose sensing and disposing. Disruption of Ca^2+^ homeostasis, which has been witnessed in the peripheral tissues and β cells of the diabetic mice and human patients, is widely considered to be a critical regulator of IR, β cell dysfunction, and T2DM.

Cellular Ca^2+^ pool encompasses several subcellular pools, primarily including the cytosolic, ER, mitochondria, Golgi apparatus, and even lysosomes ones, which intimately and dynamically communicate with each other as well as extracellular space.^387^, ^389^, ^390^ A range of extra‐ and intracellular signal stimuli could act through various signaling pathways to activate divergent Ca^2+^ channels on different membranes, resulting in Ca^2+^ influxes and thus producing specific Ca^2+^ signatures in distinct subcellular compartments.^387^, ^388^ Among them, several Ca^2+^ channels are extensively studied, such as channels voltage‐dependent Ca^2+^ channels (VDCCs) and ligand‐gated channels and transient receptor potential (TRP) channels on plasma membrane,^387^, ^388^ ORAI1 channel and sarco/ER Ca^2+^‐ATPase (SERCA) pumps on ER membrane,^391^ and voltage‐dependent anion channels (VDACs)^387^, ^389^, ^392^ and mitochondrial calcium uniporter complex on the inner and outer mitochondrial membranes.^392^, ^393^, ^394^, ^395^ Elevated Ca^2+^ concentration could initiate multiple signaling cascades by interacting with and activating the Ca^2+^ sensor protein calmodulin (CaM), which subsequently induces numerous downstream targets in various ways. The major downstream targets of Ca^2+^‐bound CaM are Ca^2+^/calmodulin‐dependent protein kinases (CaMKs), which in turn elicit the phosphorylation of CERB or the activation of calcineurin (CaN)/NFAT1 pathway,^396^, ^397^ thereby regulating glucose sensing, insulin exocytosis, and insulin transcription.^398^ In addition, many other metabolism‐related pathways and transcription factors are tightly regulated by the Ca^2+^ signaling, such as MAPK, nuclear factor‐κB (NF‐κB), myocyte enhancer factor 2, and FoxO1.^399^, ^400^, ^401^ Meanwhile, to avoid overflow of Ca^2+^ and cytotoxicity, other signaling pathways, such as the PLC/PIP2/IP3 pathway, could trigger various pumps and exchangerfFs, including the Na^+^/Ca^2+^ exchanger,^392^, ^402^ the plasma membrane Ca^2+^ ATPase,^389^, ^391^ IP3 receptor (IP3R), and ryanodine receptor (RyR),^387^, ^389^ to remove Ca^2+^ outward, thus restoring Ca^2+^ concentrations. Additionally, mitochondria‐associated ER membranes (MAMs) hinge on an IP3R1/GRP75/VDAC1 complex‐dependent mechanism to take charge of Ca^2+^ transport from the ER to mitochondria.^403^ Besides, the Golgi apparatus and lysosomes also participate in Ca^2+^ homeostasis by acting as Ca^2+^ stores, which have been elaborately reviewed elsewhere.^404^ Cumulatively, attributed to this subtle regulatory network, cellular Ca^2+^ concentrations remain lively balanced, which is central to the fine progressing of many cellular behaviors and states.

Actually, previous studies suggested that hyperglycemia relies on a Ca^2+^‐dependent mechanism to activate glucose transport in skeletal muscle.^405^ Additionally, it is well established that insulin stimulation induces a rapid and transient increase in cytoplasmic and mitochondrial Ca^2+^ through RyR and IP3R activation, which is required for GLUT4 translocation and glucose uptake.^406^, ^407^ Ca^2+^ signals also play important roles in controlling insulin secretion^408^, ^409^ and β cell proliferation.^410^, ^411^, ^412^ For example, during GSIS, fusion of insulin granules with the plasma membrane requires an increase in intracellular Ca^2+^.^413^ Consistent with the essential role of Ca^2+^ in insulin action and secretion, it was defined that Ca^2+^ dysregulation is linked to the progression of T2DM.

Dysregulation of ER Ca^2+^ concentration is notably implicated in the progression of IR in peripheral tissues. For instance, SERCA2b has been widely observed to be dramatically reduced in the liver,^414^ WAT,^415^ and skeletal muscle^416^ from obese and diabetic mice, causing the reduction of ER luminal Ca^2+^ concentration and therefore aggravating IR.^417^, ^418^ Consistently, liver‐,^417^ adipocyte‐,^415^ or muscle‐specific^419^ restoring SERCA in obese and diabetic mice could improve systemic insulin sensitivity and glucose homeostasis, ameliorate hepatosteatosis, reduce adipose mass, but increase BAT energy expenditure and UCP1/3 expression, in which reduced ER stress and decreased PKCδ activation may contribute to these observed effects. Reduced intracellular and ER Ca^2+^ storage capacity, resulting from the accumulation of cholesterol,^420^, ^421^, ^422^ has also shown to be linked to PKC activation,^423^, ^424^ as well as the development of hepatic IR.^425^, ^426^ Mechanistically, activated PKC may mediate the phosphorylation of ORAI1 and inhibit its activity, thereby decreasing the refilling of the ER Ca^2+^ stores and further aggravating hepatic IR.^427^ Of note, it has been reported that ER‐mitochondria interactions and interorganelle Ca^2+^ exchange are reduced in high‐fat high‐sucrose diet‐induced obese mice, while a healthy diet could effectively restore the communication between ER and mitochondria that improves hepatic insulin sensitivity and glucose homeostasis,^428^ which might depend on the activation of PKCε.^429^

Obesity and diabetic conditions bring about mitochondria Ca^2+^ turbulence and thus resulting in mitochondria dysfunction,^430^, ^431^ which collectively aggravate T2DM progression by abolishing insulin signaling.^430^, ^431^, ^432^ For example, obesity is reported to drive the increased localization of IP3R1 at the MAMs, leading to an overload of mitochondrial Ca^2+^ influx, grievous oxidative stress and IR in the liver.^433^, ^434^ Furthermore, disruption of MAMs and mitochondrial Ca^2+^ have been viewed as early events preceding mitochondrial dysfunction and IR in the liver and skeletal muscle.^430^, ^432^ For example, in mice fed a HFD, the mitochondrial dysfunction arising from the downregulation of glucose‐regulated protein 75 (GRP75), an important regulator for mitochondrial Ca^2+^ homeostasis, can aggravate systemic IR, while induction of GRP75 in mice could inverse such effects.^435^ Furthermore, reduced mitochondrial Ca^2+^, resulting from the mutation and acute deletion of seipin, a protein modulating mitochondrial Ca^2+^ by interacting with SERCA,^436^, ^437^ has been pointed out to impair tricarboxylic acid cycle cycles and subsequent reduction in citrate,^438^ leading to the defects in lipid storage and lipogenesis. Similar to mitochondrial Ca^2+^ deficiency, mitochondrial Ca^2+^ overload caused by excessive MAM content has also been shown to induce IR and fat deposition in hepatocytes.^439^, ^440^ Meanwhile, in the skeletal muscle, MAM formation is also found to be augmented by obesity in a PDK4‐dependent way, thereby leading to increased mitochondrial Ca^2+^ accumulation, mitochondrial dysfunction, ER stress, and consequent IR.^434^

Furthermore, other regulators and signaling pathways responsible for whole‐cellular Ca^2+^ homeostasis also play critical roles in regulating peripheral IR progression. For instance, obesity can upregulate CaMKII activity via enhancing ER stress,^441^, ^442^ which further compromises the insulin signaling in the liver.^228^ While, abolishing CaMK1D significantly improves peripheral insulin sensitivity and glucose control in DIO or HFD‐fed mice by reprograming glyceraldehyde 3‐phosphate dehydrogenase‐/peroxisomal biogenesis factor 3 (PEX3)‐mediated metabolic processes.^443^ Besides, the capsaicin‐activated TRPV1 is repressed in the adipose tissue from HFD‐fed mice,^444^, ^445^ and involved in CaMKII/AMPK/SIRT1/PPARγ‐mediated WAT browning.^444^ Moreover, activation of TRPV1 may also contribute to enhanced metabolic function of BAT,^445^ possibly through mediating SIRT1/PPARγ axis,^445^ as well as controlling clock gene oscillations, indicating a protective role of TRPV1 in metabolic diseases.^446^ However, these studies might be challenged by other results that genetic deletion or pharmacological inhibition of TRPV1 protected mice from obesity, IR, hypertension, inflammation, or leptin resistance.^447^, ^448^ Additionally, another TRP channel, TRPM2, is able to mediate angiotensin II‐induced IR in adipocytes.^449^ Moreover, silencing of herpud1, an ER membrane protein maintaining intracellular Ca^2+^ homeostasis under stress conditions, has an ability to decrease insulin‐dependent glucose uptake, GLUT4 translocation, and AKT activation in myotubes via increasing the IP3R‐dependent cytosolic Ca^2+^ response and CaN activity.^450^ Accordingly, correcting the SR lipid composition and thus Ca^2+^ handling in the skeletal muscle can lead to an improvement in IR in DIO mice.^451^

During the pathogenesis of T2DM, Ca^2+^ homeostasis in the pancreatic β cells is also discerned to be broken, which in turn aggravates the dysfunction of β cells in terms of their insulin secretion and fate decision. For example, due to reduced transcription,^408^, ^409^ changed location^452^ and depressed activity of VDCCs,^453^, ^454^ Ca^2+^ influx across VDCCs is decreased in the diabetic β cells, leading to reduced Ca^2+^ influx near insulin granules docked sites, inhibited fusion of insulin granule with the plasma membrane, and thus impaired GSIS.^452^ Specifically, it is reported that IR can induce a reduction in PIP2, and impair VDCC activity and subsequent insulin secretion.^453^ Furthermore, depletion of other critical Ca^2+^ signaling mediators, including CaMKII, CREB, CaM, CaN, and CRTC2, in mouse β cells, can impair insulin secretion and systemic glucose homeostasis.^455^, ^456^, ^457^ On the contrary, specific deletion of Ca^2+^/CaM‐dependent serine protein kinase in mouse β cells results in reduced blood glucose, hyperinsulinemia, and IR in HFD‐fed mice. Short‐term stimulation of Ca^2+^ signaling pathways might yield positive effects for β cells, whereas chronic Ca^2+^ stimulation instead presents deleterious effects on β cell mass. Transient activation of CaMKIV/CREB,^458^, ^459^, ^460^ CaN/NFAT,^461^ and CaN/TFEB^462^ pathways promote glucose‐mediated β cell proliferation and survival by reprogramming gene transcription, consistent with the positive roles of WNT/Ca^2+^ pathways in β cell survival. However, mice overexpressing constitutively active CaN or CaMKIIα in β cells instead, develop glucose intolerance and diabetes with decreased β cell mass,^463^ which is due to increased apoptosis and decreased proliferation of β cells. Furthermore, aberrantly elevated cytosolic Ca^2+^ is also required for β cell de‐ or trans‐differentiation processes, resulting in the exacerbation of T2DM.^464^ One notable example is that overexpression of CaM caused the trans‐differentiation of β cells into glucagon‐expressing cells and thus T2DM progression,^464^ while blocking β cell depolarization or Ca^2+^ influx markedly reduced trans‐differentiation of β cells into gastrin‐expressing cells in both diabetic mice and isolated islets.^465^ Additionally, β cell deletion of ATP binding cassette subfamily C member 8 (ABCC8), a subunit of the ATP‐sensitive potassium (K_ATP_) channel, results in increased cytosolic Ca^2+^ and loss of β cell maturation, accompanied by enhanced expression of dedifferentiation marker aldehyde dehydrogenase 1 family member A3 (ALDH1a3).^466^

### 4.12 Other pathways

#### 4.12.1 PPARs pathway

PPARs, including PPARα, PPARβ/δ, and PPARγ, have been characterized as core lipid sensors that modulate whole‐body energy metabolism (Figure 9).^467^ PPARα is the predominant PPAR isoform in the liver and plays an important role in regulating fatty acid transport, ketogenesis, and β‐oxidation.^468^ In addition to lipid regulation, while, PPARβ/δ could increase the production of GLP‐1 to stimulate insulin secretion and reduce IR.^469^ PPARγ mainly functions as a core regulator of adipogenesis, improving insulin sensitivity and glucose metabolism. In terms of mechanisms, deletion of IRF3 can increase the expression of PPARγ, leading to severe IR and glucose intolerance.^470^ As opposed to IRF3, TAZ could protect mice from HFD‐induced IR and glucose intolerance through upregulating the expression of PPARγ.^471^ Furthermore, PPARγ may induce the enhanced level of FGF1 in the adipose tissue, and considering the aforementioned protective function of FGF1 in insulin sensitivity and glucose metablism,^363^ PPARγ/FGF1 axis may be indispensable in maintaining insulin sensitization an metabolic homeostasis.^364^

#### 4.12.2 SIRTs pathway

As highly conserved NAD^+^‐dependent protein deacetylases,^472^ SIRTs are composed of seven subtypes and have been shown to regulate insulin secretion and affect hepatic insulin signaling and glucose homeostasis. SIRT3 expression is significantly decreased in the islets of T2DM patients and diabetic mice, which contributes to increased ROS level, mitochondrial dysfunction, and impaired insulin secretion.^473^ Furthermore, SIRT1 could repress the transcription of UCP2, which promotes the conversion from glucose to ATP and Ca^2+^ influx, thus initiating insulin secretion and augmenting the sensitivity of pancreas to glucose.^474^ What's more, SIRT1 and SIRT6 may deacetylate PGC1α and FoxO1 to facilitate the transcription of gluconeogenic genes,^475^, ^476^ ultimately regulating GSIS in the islets^477^ and ameliorating glucose homeostasis in the liver. Of note, SIRT1 could also react with PPARγ to reduce the activity of PPARγ in WAT, thus inhibiting insulin signaling and adipogenesis.^478^

#### 4.12.3 IL‐6 pathway

By binding to membrane receptors, IL‐6 leads to the activation of multiple downstream signaling pathways inside target cells, such as PI3K/AKT, MAPK, and janus kinase (JAK)/STAT.^479^ IL‐6 is reported to inhibit IRS, AKT2 and ERK phosphorylation directly or indirectly in the liver, leading to impaired insulin signaling pathway and IR.^480^, ^481^, ^482^, ^483^ Besides, IL‐6 is able to suppress glucose transport by affecting adiponectin and GLUT4,^484^ repress lipogenesis by inhibiting PPARγ,^485^ and stimulates lipolysis by activating AMPK in the adipose tissue,^486^ which may link the IL‐6 pathway to the progression of IR and T2DM. However, there are also some conflicting results. For instance, in the case of HFD feeding, deletion of IL‐6 and IL‐6R promoted the development of hepatic IR.^487^, ^488^ In addition, the role of IL‐6 in pancreatic islets appears to be contradictory. Several studies have shown that IL‐6 contributes to the induction of low grade inflammation in the islets, which ultimately results in impaired insulin secretion.^489^, ^490^, ^491^ Paradoxically, acute exposure of IL‐6 does not seem to exert its potential hazardous effects on the islets,^492^ or affect the normal functioning of β cells.^480^, ^493^, ^494^, ^495^

#### 4.12.4 JAK/STAT pathway

Depending on the cytokine or growth factor signals, different combinations of JAKs and STATs are activated with a high degree of specificity. Multiple JAK/STAT pathways have been implicated in glucose metabolism, including the IL‐6/STAT3, GH/JAK2/STAT5/IGF1, and IL‐4/STAT6 axes. Specifically, liver‐specific knockout of STAT3 in mice blocked the IL‐6 signaling pathway, leading to increased IR and gluconeogenesis,^496^, ^497^ via the induction of toll‐like receptor 4 expression.^498^ Knockout of the genes encoding growth hormone receptor, JAK2 or STAT5 in the liver and muscle also enhanced lipid accumulation and IR,^499^, ^500^ possibly due to increases in FFAs flux and DNL.^500^ However, the role of JAK2/STAT5 in the adipose tissue still remains controversial.^501^, ^502^ What we do know is that the IL‐4/STAT6 pathway has been shown to increase glucose oxidation by inhibiting PPARα activity in hepatocytes, while knockout of STAT6 promotes hepatic steatosis and IR.^503^

The JAK/STAT pathway also plays a role in lipid metabolism. Mice with adipose‐specific *jak2* knockout had impaired lipolysis in response to growth hormones and leptin.^501^, ^504^ Likewise, loss of either STAT3 or STAT5^505^, ^506^ significantly impaired lipolysis, probably resulting from repression of essential lipolytic genes. Muscle‐specific deletion of *stat5* leads to increased accumulation of lipids in skeletal muscle, dyslipidemia, hepatic steatosis, and hyperglycemia, accompanied by altered expression of genes involved in lipogenesis, lipid uptake, lipolysis, insulin signaling, and glucose uptake.^507^

#### 4.12.5 NLRP3 inflammasome

The NOD‐like receptor (NLR) family pyrin domain‐containing 3 (NLRP3) inflammasome is a cytosolic multiprotein complex composed of the innate immune receptor NLRP3, the adapter apoptosis‐associated speck‐like protein containing card (ASC), and the inflammatory protease caspase‐1. Several endogenous ligands related to metabolic stress, such as lipopolysaccharide (LPS),^508^, ^509^ palmitic acids, oleic acid,^510^ homocysteine and ROS,^511^, ^512^ have been reported to activate NLRP3 inflammasome in metabolic organs. Activated NLRP3 inflammasome induces the maturation and release of IL‐1β and IL‐18, responding to microbial infection, endogenous danger signals, and environmental stimuli.^513^ IL‐1β has been demonstrated to induce IR by reducing IRS1 phosphorylation and expression^514^ and inducing IL‐6 release.^515^ The deficiency of NLRP3, caspase‐1, or IL‐1β in the liver and adipose tissue can result in reduced inflammasome activation, improved lipid metabolism, enhanced insulin sensitivity, and reduced blood glucose in DIO mouse models.^516^, ^517^, ^518^ Additionally, IL‐1β can influence the activation of MAPK^519^ and NF‐κB,^520^ which in turn dysregulates the expression of genes involved in β cell death. Likewise, NLRP3 knockout is able to protect β cells from damage, rescue HFD‐induced β cell loss, and increase insulin secretion.^521^, ^522^

#### 4.12.6 NF‐κB pathway

NF‐κB is an important transcription factor controlling different biological processes, such as immune activation, cell survival and stress response.^523^ In canonical NF‐kB signaling, cytokines and PAMPs stimulate cell surface receptors to initiate activation of the inhibitory κB proteins (IκB) kinase (IKK) complex composed of IKK1 (IKKα) and IKK2 (IKKβ) and the regulatory subunit NEMO (IKKγ), ultimately driving transcription of target genes. IKKβ has been shown to phosphorylate and inactivate TSC1 in the mTOR pathway,^524^ as well as IRS,^525^ and S6K1^526^ in the PI3K/AKT pathway, to promote IR. In both genetic‐ and DIO mice, deficiency of IKKB can prevent the advancement of IR and glucose intolerance.^527^, ^528^ Besides, NF‐kB may drive the expression of a plethora of inflammatory chemokines and cytokines in multiple metabolic tissues^529^ and regulate numerous genes involved in insulin signaling, such as SOCS3 and protein tyrosine phosphatase 1B (PTP1B).^530^, ^531^ In the pancreas, inhibition of IKKβ is sufficient to provide protection against cytokine‐induced β cell apoptosis in both human and mouse diabetic islets,^532^ thus regulating β cell mass and insulin secretion.^533^

## 5 THERAPEUTIC TARGETS IN T2DM COMPLICATIONS

**FIGURE 10:**
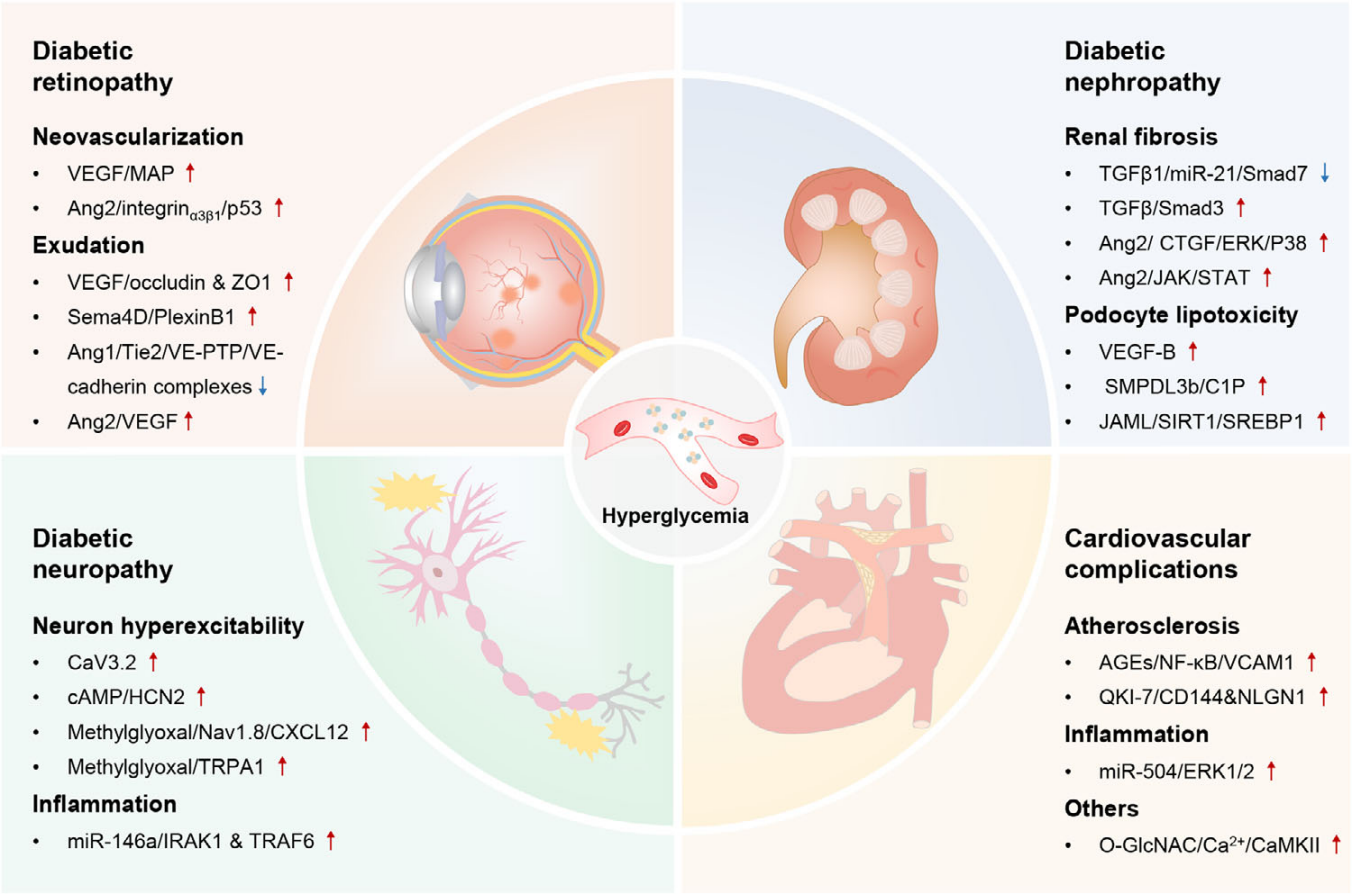
Signaling pathways in T2DM complications. Hyperglycemia causes macrovascular and microvascular lesions, contributing to diabetic complications such as diabetic retinopathy, diabetic nephropathy, diabetic neuropathy, and diabetic cardiovascular disease. Targeting the major pathological features of different complications, some signaling pathways have been discovered. These signaling pathways play important roles in promotion or inhibition of disease development, contributing to the treatment of diabetic complications.

T2DM causes a number of long‐term complications in the neural, macrovascular and microvascular systems, which are instrumental for death and disability of patients with T2DM (Figure 10).^10^, ^534^ These complications encompass the damages to the coronary and cerebrovascular arteries, the kidneys, the eyes, and the nerves.^10^, ^534^ In the following section, we will revisit the potential signaling pathways, as well as their associated therapeutic targets, underlying T2DM complications.

### 5.1 Diabetic nephropathy

Diabetic nephropathy is perhaps among the most devastating T2DM complications, which bedevils up to 40% of T2DM patients and leads to kidney failure, CVD, and premature death.^535^ The pathological changes of DN mainly include renal fibrosis and podocyte injury,^536^ which are linked to various signaling pathways, particularly the TGFβ and mTOR pathways.

Dysregulation of TGFβ pathway is widely considered playing a key role in renal fibrosis, which is characterized by excessive deposition of ECM. Correspondingly, targeting TGFβ was also identified to be of therapeutic potential for DN.^537^, ^538^ Considerable evidence has revealed increased expression and activation of TGFβ pathway components, such as TGFβ1 and SMAD2/3, in the fibrotic kidney.^537^, ^538^ Consistently, inhibition of TGFβ1 or its downstream pathways substantially restrained renal fibrosis in DN, whereas overexpression of TGFβ1 prompted renal fibrosis, suggesting a profibrotic role of TGFβ pathway in DN.^537^, ^538^ TGFβ pathways can induce the activation of myofibroblasts, apoptosis of endothelial cells and podocytes, overproduction of ECM, and impairment of ECM degradation via multiple downstream mechanisms.^538^, ^539^, ^540^, ^541^ These downstream mechanisms include the imbalance between upregulation of profibrotic SMAD3 and downregulation of antifibrotic SMAD7, interplays with other signaling pathways (e.g., MAPK, WNT/β‐catenin, ERK, PI3K/AKT, mTOR pathways), and the involvement of miRNAs, lncRNAs and epigenetic modifications.^538^ Accordingly, numerous approaches blocking TGFβ signaling, including TGFβ neutralizing antibodies, antisense TGFβ oligodeoxynucleotides, soluble human TGFR2, and specific inhibitors to TGFR1 kinases can effectively halt the progression of renal fibrosis and DN.^537^, ^538^, ^542^ However, due to the side‐effects of TGFβ inhibition in autoimmune diseases, direct targeting of TGFβ1 is barely possible to be a viable strategy against renal fibrosis and DN.^538^ Alternatively, indirect approaches to antagonizing TGFβ signaling might hence be promising to yield effective therapeutic agents for DN. In addition to TGFβ signaling, other signaling pathways may be of therapeutic potential in renal fibrosis and DN. For example, it has shown that high glucose can trigger the JAK/STAT signaling cascade to stimulate excessive proliferation of glomerular mesangial cells and aggravate DN,^543^ and the small‐molecule inhibitor of JAK/STAT, baricitinib, has been demonstrated to decrease albuminuria in patients with T2DM and DN.^544^

Podocyte injury is another important pathological feature of DN, in which the mTOR pathway is hyper‐activated due to high concentrations of glucose.^545^, ^546^ Enhanced mTORC1 signaling may trigger podocyte injury and DN by stimulating podocyte hypertrophy, mesangial expansion, glomerular basement membrane thickening, foot process effacement, podocyte loss, and albuminuria.^546^, ^547^ In line with this, restoration of mTORC1 activity is a critical therapeutic approach for the prevention of DN. For example, rapamycin, a specific and potent inhibitor of mTOR, markedly ameliorated mesangial expansion and proteinuria.^548^ Moreover, the mechanism by which metformin and SGLT2 inhibitors exert kidney‐protective effects may be also related to the mTOR pathway, albeit it is currently unclear.^546^

### 5.2 Diabetic retinopathy

Nearly 60% of T2DM patients are expected to have DR in the first decade after diagnosis.^549^, ^550^ DR is characterized by early pericyte loss, vascular leakage, retinal ischemia, and an overcompensatory retinal neovascularization, which is highly related to the regulation of signaling pathways.^551^

Due to the local ischemia/hypoxia, the expression of VEGF is enhanced in ocular fluid of patients with DR.^552^, ^553^, ^554^ Upregulated VEGF can increase the phosphorylation of occludin/zonula occludens‐1 (ZO‐1) to augment endothelial paracellular permeability, and cause activation of MAP to promote the proliferation of endothelial cells, thereby exacerbating the development of DR.^555^, ^556^ In parallel, intravitreal injection neutralizing antibody of VEGF, such as Ranibizumab, Pegaptanib, and Aflibercept, has been clinically used to treat proliferative diabetic retinopathy.^550^, ^557^, ^558^

As endogenous ligands for the vascular endothelial receptor tyrosine kinase 2 (Tie2), angiopoietin1/2 (Ang1/2) are involved in the regulation of vascular permeability and angiogenesis in DR.^559^ Current evidence showed that Ang1 promotes the formation of Tie2/PTP/VE‐cadherin complexes at cellular membrane to decrease vascular permeability,^560^ but Ang2 enhances vascular leakage in DR through activating VEGF/β1‐integrin and regulating VE‐cadherin‐containing cell‐cell junctions.^561^, ^562^, ^563^ Meanwhile, Ang2 could reduce pericyte capillary coverage and increase intraretinal neovascularization via the inhibition of Ang1/Tie2 interactions, suggesting that modulation of Ang1/2 is an attractive therapeutic target for the prevention and treatment of DR.^564^

Emerging evidence suggests an essential role of circular RNAs (circRNAs) and miRNAs in DR.^565^, ^566^ For example, diabetes‐related stress upregulates the expression of cPWWP2A, a novel circRNA, to sequester and inhibit miR‐579 activity, thus alleviating T2DM‐induced retinal vascular dysfunction.^565^ cZNF532 is another circRNA that could regulate pericyte biology by sequestering miR‐29a‐3p and activating chondroitin sulfate proteoglycan 4 (CSPG4), lysyl oxidase like 2 (LOXL2), and CDK2, which indicates that overexpression of cZNF532 or inhibition of miR‐29a‐3p could ameliorate DR.^567^

### 5.3 Diabetic neuropathy

Among diabetes neuropathy, distal symmetric polyneuropathy is very common and defined as a loss of sensory function beginning distally in the lower extremities.^568^ Approximately 30–50% of patients with diabetic neuropathy develop neuropathic pain called painful diabetic neuropathy (PDN).^569^ PDN is characterized by neuropathic pain, small‐fiber degeneration, and dorsal root ganglion (DRG) nociceptor hyperexcitability.^570^

DRGs are important neurons that comprise thermoreceptors, mechanoreceptors, and itch sensors. Nav1.8, an α‐subunits of voltage‐gated sodium channels, is important for the development of abnormal pain sensation.^571^ Methylglyoxal, a reactive metabolite increased in diabetes, could posttranslationally modify Nav1.8 and enhance the activity of the nonselective cation channel TRPA1, respectively resulting in Nav1.8 gain of function pain and neuron hyperexcitability.^572^, ^573^ In addition, excitatory CXCR4/CXCL12 signaling in Nav1.8‐positive DRG neurons plays a critical role in the pathogenesis of mechanical allodynia and small‐fiber degeneration in a mouse model of PDN.^570^ In T2DM, Ca^2+^ channels have also been implicated in PDN. For example, CaV3.2 activity is enhanced through the glycosylation of extracellular arginine residues, resulting in hyperexcitability pain in DRG neurons.^574^

### 5.4 Cardiovascular complications

Macrovascular complications of T2DM include coronary heart disease, cardiomyopathy, arrhythmias and sudden death, cerebrovascular disease, and peripheral artery disease, among which CVD is the primary cause for death in diabetic patients. The development of diabetic cardiovascular complications also relates to the dysregulation of signaling pathways. A prominent example is that hyperglycemia can induce the formation of nonenzymatically glycated proteins or lipids, called advanced glycation end‐products (AGEs), which is closely related to the pathogenesis of CVD. AGEs have been shown to activate NF‐κB and increase vascular cell adhesion molecule1 (VCAM1) expression, initiating the first step of atherogenesis.^575^ Besides, glycated low‐density lipoprotein (LDL) is recognized and taken up by AGE receptors or macrophage scavenger receptors, resulting in lipid‐laden foam cells in the arterial intima and the promotion of atherosclerosis.^576^ In vascular smooth muscle cells (VSMC) and aortas from db/db mice, miR‐504 might participate in metabolic memory of CVD,^577^ as evidenced by that miR‐504 in VSMC can inhibit contractile genes and enhance the activation of ERK1/2 and its target genes (Grb10 and Egr2), inhibiting proinflammatory response. Additionally, quaking (QKI) is one of the RNA‐binding proteins related to diabetic cardiomyopathy and atherosclerosis. QKI‐7 is highly expressed in human coronary arterial ECs in T2DM patients and is able to disrupt cell barrier, compromise angiogenesis, and enhance monocyte adhesion by binding and promoting mRNA degradation of downstream targets.^578^

### 5.5 T2DM and COVID‐19

Recent evidence showed a mutual interplay between COVID‐19 and T2DM.^579^ On the one hand, COVID‐19 patients with diabetes are more likely to develop severe condition of higher death incidence compared with those without diabetes.^580^, ^581^ On the other hand, new‐onset hyperglycemia, ketoacidosis, diabetes, and severe metabolic complications of preexisting diabetes have been observed in patients with COVID‐19.^582^, ^583^, ^584^ There is an intricate interaction network existing between diabetes and COVID‐19. Common pathogenetic processes between COVID‐19 and diabetes mellitus discovered by differential gene expressions pattern analysis are related to the mRNA metabolism, subcellular organelle organization, nucleotide synthesis, immune responses, and autophagy process. Other studies revealed that five biomarker genes (*CP*, *SOCS3*, *AGT*, *PSMB8*, and *CFB*) and 4 miRNAs (hsa‐miR‐298, hsa‐miR‐3925‐5p, hsa‐miR‐4691‐3p, and hsa‐miR‐5196‐5p) closely bridge T2DM and COVID‐19.^585^, ^586^, ^587^

Severe COVID‐19 is associated with worse glycemic control,^588^ and the reason for this may partly lie in increased expression of ACE2, a key receptor for severe acute respiratory syndrome coronavirus‐2 (SARS‐CoV‐2), in lungs of T2DM patients.^589^ Growing evidence suggests that elevated glucose level and glycolysis may directly increase and sustain SARS‐CoV‐2 replication.^590^ Meanwhile, diabetes can affect immune response to SARS‐CoV‐2. Hyperglycemia and IR are capable of increasing synthesis of AGEs and proinflammatory cytokines to mediate tissue inflammation.^591^ Additionally, hyperglycemia may also impede type I interferon production and signaling^591^ to augment inflammatory responses to SARS‐CoV‐2 infection, leading to extreme systemic immune responses.^592^, ^593^

The precise mechanisms underlying the development of new‐onset T2DM in people with COVID‐19 are still not known, but may involve a number of complex etiologies. On the one hand, SARS‐CoV‐2 could directly infect β cells and lead to β cell dysfunction and metabolic dysregulation.^594^ SARS‐CoV‐2 could also bind to NRP1, thus attenuating insulin secretion and inducing β cell apoptosis through the JNK/MAPK pathway.^595^ Meanwhile, SARS‐CoV‐2 might downregulate ACE2 activity and subsequently activate macrophages and trigger NF‐κB signaling in the pancreas, leading to excessive synthesis and secretion of inflammatory cytokines, eventually damaging islets and β cells.^596^, ^597^ On the other hand, IR, resulting from viral infection seems to be another cause of hyperglycemia upon COVID‐19. Infection of SARS‐CoV‐2 may induce an inflammatory state,^598^, ^599^ in which IL‐6 might play a primary albeit not exclusive role in inducing IR and β cell dysfunction.^579^, ^600^

## 6 ANTIDIABETIC DRUGS

**FIGURE 11:**
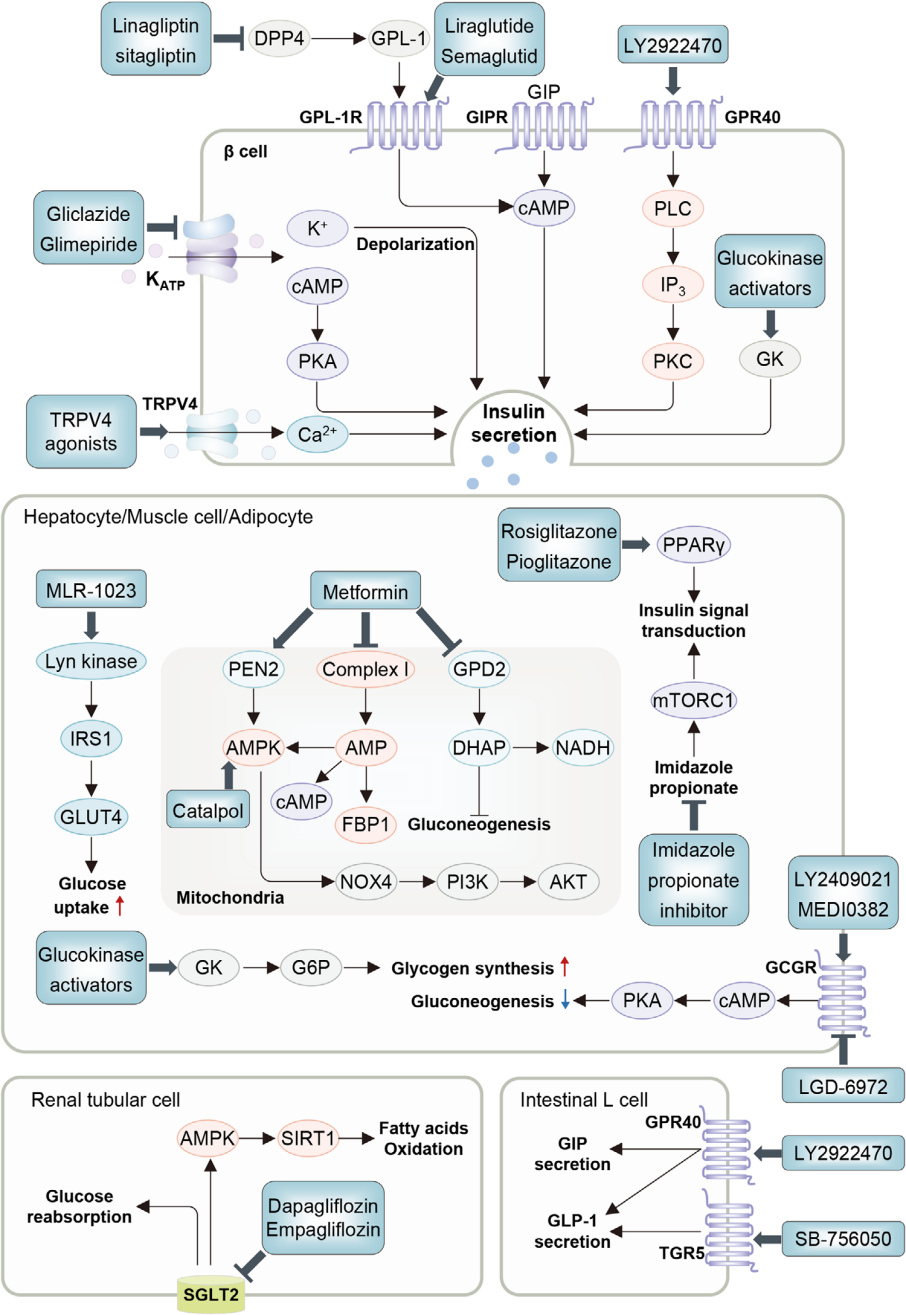
Clinical and potential drugs in T2DM therapy. In the plasma, DPP4 inhibitors, such as Linagliptin, increase the plasma content of GLP‐1 by inhibiting the degradation of GLP‐1 by DPP4. In pancreatic β cells, the agonists of GLP‐1R (GLP‐1, Liraglutide), GIPR, and GPR40 (LY2922470) promote insulin secretion through different signaling pathways. The inhibitor of K_ATP_ (Gliclazide) and the agonist of Ca^2+^ channel also promote the secretion of insulin. In metabolic organs, MLR‐1023 activates the Lyn kinase, leading to tyrosine phosphorylation of IRS1. Then a cascade of signaling events lead to increased glucose uptake and utilization. Metformin could bind to PEN2 to forms a complex with ATP6AP1, a subunit of the v‐ATPase8, leading to the inhibition of v‐ATPase and the activation of AMPK. Inhibition of the mTORC1/2 pathway may enhance insulin signaling at the IRS level and improve glucose tolerance. The activation of glucagon receptor results in increased glycogenolysis and gluconeogenesis via cAMP/PKA pathway. In the kidney, Ertugliflozin and Dapagliflozin promote urinary glucose excretion by inhibiting SGLT2. In the intestine, the agonist of TGR5 (SB‐756050) and GPR40 (LY2922470) promotes the secretion of GIP and GLP‐1.

Poor control of hyperglycemia results in markedly increased microvascular, macrovascular and metabolic complications, and therefore, optimal glycemic management is the first priority in T2DM patients.^601^ Although diet control and exercise could partly alleviate hyperglycemia, almost all T2DM patients still need antidiabetic drugs and/or insulin to maintain standard blood glucose. With the in‐depth study of T2DM signaling pathways, effective glucose‐lowering drugs often work by acting on important targets in signaling pathways. In the following section, we will summarize glucose‐lowering drugs in clinical application and clinical trials, as well as promising agents and targets, describing their action mechanisms and related signaling pathways (Figure 11 and Table 1).

**TABLE 1:** Agents for T2DM in clinical or clinical trials

Agent	Target or pathway	Phase	NCT number
Metformin hydrochloride	AMPK/PI3K/AKT/mTOR; autophagy; mitophagy	US FDA approved	NA
Ertugliflozin	SGLT2	US FDA approved	NA
Dapagliflozin		US FDA approved	NA
Chlorpropamide	Na^+^ K^+^‐ATPase	US FDA approved	NA
Acarbose	Glucosidase inhibitor	US FDA approved	NA
Voglibose		US FDA approved	NA
Miglitol		US FDA approved	NA
Saxagliptin	DPP4	US FDA approved	NA
Linagliptin		US FDA approved	NA
Alogliptin		IV	NCT03042325
Trelagliptin		IV	NCT02771093
DBPR108		III	NCT04218734
Retagliptin		III	NCT05054842
Gliclazide	K^+^ channel	US FDA approved	NA
Glipizide		US FDA approved	NA
Glyburide		US FDA approved	NA
Glimepiride		US FDA approved	NA
Repaglinide		US FDA approved	NA
Mitiglinide		IV	NCT02143765
MSDC‐0160		II	NCT00760578
Liraglutide	GLP‐1R	US FDA approved	NA
Semaglutide		US FDA approved	NA
Taspoglutide		III	NCT00909597
Pioglitazone	PPAR	US FDA approved	NA
Rosiglitazone		US FDA approved	NA
FK614		II	NCT00036192
LY3298176	GLP‐1R/GIPR	III	NCT03882970
AZD1656	Glucokinase	II	NCT01152385
Dorzagliatin		II	NCT04531631
MK‐0941		II	NCT00824616
PF‐04937319		II	NCT01475461
LGD‐6972	GCGR	II	NCT02851849
LY2409021		II	NCT01241448
PF‐06291874		II	NCT02554877
Cotadutide	GLP‐1R/GCGR	II	NCT04208620
MEDI0382		II	NCT03745937
SAR425899		II	NCT02973321
GSK1292263	GPR119	II	NCT01119846
GSK256073	GPR109A	II	NCT01376323
MLR‐1023	Insulin receptor	II	NCT02317796
AZD7687	Acyltransferase	I	NCT01217905
LY2922470	GPR40	I	NCT01746017
MK‐8666		I	NCT01971554
LY2405319	FGF21 receptor	I	NCT01869959
SB756050	Bile acid receptor	I	NCT00607906
Coenzyme Q	Electron transport chain	II	NCT00703482
MitoQ		NA	NCT04558190

This table summarizes agents used to treat diabetes by targeting different signaling pathways, in clinical and preclinical.

*Data source*: U.S. National Library of Medicine *ClinicalTrials.gov*.

### 6.1 Clinical drugs

#### 6.1.1 Biguanides

As a first‐line drug for T2DM, metformin has been shown to have pleiotropic effects on glucose metabolism.^602^ The core glucose‐lowering effect of metformin is the inhibition of hepatic gluconeogenesis by repressing mitochondrial respiratory chain complex 1 and increasing AMP level.^603^, ^604^ In addition, metformin can bound to PEN2 and form a complex with ATP6AP1 to inhibit v‐ATPase and activate AMPK.^15^ Metformin can also directly act on the intestine to activate muscarinic M3 receptor, WNT and AMPK to stimulate GLP‐1 secretion, thereby altering glucose absorption.^602^ Additionally, metformin could prevent the reabsorption of active BAs by modulation of the transcription of FXR via an AMPK‐mediated mechanism in enterocytes, ultimately increasing GLP‐1 secretion.^605^ Moreover, metformin has beneficial effects on alleviating chronic low‐grade inflammation in T2DM.^606^ It not only inhibits monocyte‐to‐macrophage differentiation via inhibiting STAT3,^607^ but also suppresses LPS‐induced inflammatory response in macrophages via the AMPK/ATF3 pathway.^608^

#### 6.1.2 PPARγ agonists

Thiazolidinediones (TZDs), a kind of classic glucose‐lowering drugs, are the ligands for PPARγ. PPAR agonists primarily stimulate WAT remodeling and modulate lipid flux to improve insulin signaling and glucose homeostasis. TZDs binding to PPARγ could promote FFAs storage and reduce lipid ectopic accumulation in subcutaneous fat tissue.^609^ Rosiglitazone and pioglitazone are the representatives of TZDs. Due to the potential for serious adverse cardiovascular effects, the United States Food and Drug Administration adds a "black box warning" to the rosiglitazone label,^610^ while pioglitazone can significantly lower risk of death, myocardial infarction, or stroke among patients with diabetes.^611^ In order to make good use of PPARγ, some studies focused on the potential effects of posttranslational modifications (PTMs) of PPARγ on treating T2DM in terms of phosphorylation, acetylation, ubiquitination, SUMOylation, O‐GlcNAcylation, and S‐nitrosylation. PTMs have shed light on selective activation of PPARγ, which shows great potential to circumvent TZDs' side effects while maintaining insulin sensitization.^612^

#### 6.1.3 K^+^ channel inhibitors

Changes in membrane potential are critical for insulin secretion, and SUs are a class of drugs that can depolarize cell membrane and promote insulin secretion.^613^ By binding to the sulfonylurea receptor that is tightly linked to K^+^ channels, SUs can lead to the closure of K_ATP_ channels and the depolarization of β cell membrane, thereby stimulating insulin secretion.^614^, ^615^ In addition, SUs may enhance the GSIS by increasing intracellular cAMP level,^613^ which further activates Epac2, a protein that has an ability to exchange guanine nucleotide with Rap.^616^ Gliclazide is a representative of SUs, while Repaglinide is a representative drug in non‐SUs insulin secretagogues. With a different structure with SUs,^617^ Repaglinide binds to its receptors and closes K_ATP_ channels to depolarize β cells, inducing Ca^2+^ influx and promoting Ca^2+^‐dependent insulin granules exocytosis.^618^

#### 6.1.4 GLP‐1R agonists and DPP4 inhibitors

GLP‐1 is an incretin hormone and exerts its action by binding to GPCRs to stimulate insulin secretion through rapid increases of cAMP and intracellular Ca^2+^.^619^ In β cells, the binding of GLP‐1 and its receptor leads to activation of adenylate cyclase (AC) and a subsequent increase in cAMP. cAMP activates PKA, thereby inducing the closure of K^+^ channel and the opening of Ca^2+^. The subsequent Ca^2+^ influx promotes exocytosis of insulin granules and acute secretion of insulin into the circulation.^620^ GLP‐1 also contributes to the control of blood glucose by inhibiting glucagon secretion, gastric emptying, and food ingestion.^619^, ^621^ GLP‐1R agonists also have a role in the treatment of neurodegenerative diseases,^622^ nonalcoholic fatty liver disease^623^ and obesity.^624^ Given that GLP‐1 is mainly inactivated by dipeptidyl peptidase4 (DPP4) within 3 minutes in the circulation,^625^ DPP4 inhibitors are applied to provide prolonged effects in vivo.^626^ Notably, recent studies suggested DPP4 as a functional receptor for MERS‐CoV and SARS‐COV‐2, and its inhibitors have been found to inhibit infection with coronavirus.^627^, ^628^

#### 6.1.5 SGLT2 inhibitors

SGLT2, a high‐capacity transporter in the proximal tubule, is the major pathway for renal glucose reabsorption. SGLT2‐inhibition prevents the reabsorption of filtered glucose and sodium, resulting in glycosuria and natriuresis.^629^ SGLT2 inhibitors (SGLT2is) therefore are developed as a new class of antidiabetic drugs in T2DM, reducing plasma glucose levels in an insulin‐independent manner.^630^ Canagliflozin, a representative SGLT2i, was reported to promote mitochondrial oxidative phosphorylation and FA oxidation via the AMPK/SIRT1/PGC1α pathway.^631^ SGLT2is could also reduce cardiovascular events and protect kidney,^632^, ^633^ but increase the risk for diabetic ketoacidosis by promoting the production and reabsorption of ketone.^634^

#### 6.1.6 AGIs

Alpha‐glucosidase is an intestinal brush border enzyme responsible for the hydrolysis of disaccharides into monosaccharides, which is necessary for carbohydrate absorption.^635^ Inhibition of alpha‐glucosidase results in reduced carbohydrates absorption and increased GLP‐1, thus decreasing the postprandial blood glucose.^636^, ^637^ Alpha‐glucosidase inhibitors that are currently used in clinic include acarbose, voglibose, and miglitol, as well as a variety of natural products such as hypericin, oleanolic acid and ursolic acid.^638^

### 6.2 Preclinical drugs

#### 6.2.1 GK activators

GK elicits GSIS in β cells and promotes hepatic glycogen production and storage.^639^ Under raised glucose concentrations, GK dissociates from the GK regulatory protein, a competitive inhibitor of glucose, and phosphorylates glucose to G6P, thus reducing serum glucose and promoting HGP.^24^ Thus targeting GK may have therapeutic effects on T2DM, and several GK activators have been tested in animal experiments, in which a few have reached the clinical trials phase.^639^

#### 6.2.2 GCGR antagonists and agonists

The glucagon receptor (GCGR) is a GPCR. Its activation results in increased glycogenolysis and gluconeogenesis via the cAMP/PKA pathway,^640^ while its antagonization improves glucose control in T2DM. Nevertheless, adverse events stopped the development of GCGR antagonists (GRA). Although with new approaches, none of GRA has progressed to phase III clinical trials so far.^641^ Additionally, glucagon signaling has been associated with increased energy expenditure. GCGR agonists can increase energy expenditure and reduce hepatic fat by promoting HGP. Although this may cause a spike in blood glucose, which can be effectively offset by GLP‐1R agonists.^642^ Thus, GCGR has become a focus as a pharmaceutical target in the context of bi‐ or tri‐modal peptide agonists for the treatment of metabolic diseases.^643^

#### 6.2.3 TGR5 agonists

TGR5 agonists have been proposed as a potential treatment for T2DM.^644^, ^645^ Activated TGR5 not only stimulates GLP‐1 secretion, but also induces activity of type 2 iodothyronine deiodinase, resulting in increased thermogenesis and energy expenditure.^646^, ^647^ SB‐756050, a selective TGR5 agonist, could produce a significant enhancement of glucose‐induced GLP‐1 secretion in combination with DPP4, improve glucose disposal rate, and enhance insulin secretion.^648^ Unfortunately, SB‐756050 did not show consistent efficacy in clinical trials.^648^ Interestingly, it has recently been reported that a FXR/TGR5 dual agonist prevents progression of nephropathy in diabetes and obesity,^649^ suggesting an attractive strategy for the therapy of T2DM and its complications.

#### 6.2.4 GPR40 agonists

The G protein‐coupled receptor 40 (GPR40), also known as free fatty acid receptor 1 (FFAR1), is highly expressed in the pancreas and enteroendocrine cells in the gastrointestinal tract. When glucose level elevates, GPR40 facilitates insulin secretion through the PLC/IP3/PKC pathway.^650^, ^651^ GPR40 could also promote incretin secretion, such as GLP‐1 and gastric inhibitory polypeptide.^652^ LY2922470 is a GPR40 agonist that has shown an effective and persistent dose‐dependent reduction in glucose levels and a significant increase in insulin and GLP‐1 secretion in preclinical trials.^653^ GPR40 agonists might also regulate inflammatory responses and thus play a role in improving the development of diabetes complications.^654^, ^655^

#### 6.2.5 Lyn kinase activators

Activation of Lyn kinase can directly induce tyrosine phosphorylation of IRS1, resulting in increased GLUT4 translocation, and enhanced glucose uptake and utilization.^656^ MLR‐1023 is a highly potent and selective Lyn kinase activator, as well as a novel non‐PPARγ insulin sensitizer, which could reduce plasma glucose levels without the risk of hypoglycemia or weight gain.^656^, ^657^

#### 6.2.6 Mitochondria‐targeted antioxidants

Several mitochondria‐targeted antioxidants and peptides have been found to reduce oxidative stress and mitochondrial damage, so as to exert therapeutic effect on some chronic diseases such as diabetes and Alzheimer's disease. These antioxidants include SkQ1, MitoQ, and Coenzyme Q.^658^ Both SkQ1 and MitoQ are ubiquinone derivatives. Ubiquinone can serve as electron carriers and antioxidants to profoundly prevent lipid peroxidation.^659^ Interestingly, Coenzyme Q is a lipid‐soluble molecule, one of the key components of electron transport chain. After being oxidized, Coenzyme Q needs to undergo a self‐reducing process before it can continue to function as an antioxidant.^660^ Szeto‐Schiller (SS) peptide is a novel mitochondrial‐targeted peptide.^661^ The water‐soluble tetrapeptide SS‐31 concentrates in the inner mitochondrial membrane and may exert radical‐sweeping ability without reliance on mitochondrial membrane potential and energy.^662^ Given that administration of mitochondria‐targeted antioxidants may restore mitochondrial function and alleviate symptoms, further examination and analysis of treatment efficacy and safety are suggested to prepare for clinical trials.

### 6.3 Potential therapeutic targets and drugs

In addition to the antidiabetic drugs mentioned above, some potential targets and compounds are emerging to improve T2DM pathology. Although no effective target drugs have been developed, targeting these potential targets or modifying compounds could provide new ideas and possible therapeutic strategies for treating T2DM and its complications. For example, FGFs, especially FGF1 and FGF21, have emerged as a promising solution to the diabetes dilemma. Central injection of FGF1 can improve central glucose sensing and peripheral glucose uptake through restoring glucose‐sensing neurons, inducing neurogenesis, suppressing reactive astrocytes and restoring synaptic functionality.^344^ While FGF21 plays important roles in regulating energy balance and glucose and lipid homeostasis through a heterodimeric receptor complex comprising FGFR1 and β‐klotho.^343^ Imidazole propionate is a microbially produced histidine‐derived metabolite that impairs glucose tolerance by inhibiting insulin signaling at the level of IRS through activation of the p38γ/p62/mTORC1 pathway.^663^ Inhibition of imidazole propionate may be effective in controlling blood glucose. Similarly, β‐aminoisobutyric acid (BAIBA) is a natural catabolite of thymine. It was previously reported that BAIBA attenuated inflammation and IR in HFD‐fed mice, and these effects were negated by siRNA‐mediated suppression of AMPK.^664^ Other potential drugs/targets, such as catalpol (a natural product isolated from the root of rehmannia glutinosa), thioredoxin‐interacting protein (TXNIP, a cellular redox regulator upregulated in diabetes),^665^ and transient receptor potential vanilloid 4 (TRPV4, a Ca^2+^‐permeable nonselective cation channel)^666^ have been reported to be involved in the development of T2DM, including but not limited to affecting insulin production and secretion,^667^ β cell function,^143^ IR,^668^ and glucose homeostasis.^665^, ^669^

## 7 CONCLUSION AND PERSPECTIVES

Throughout the past decades, the surged prevalence of T2DM and its vicious complications has coined an urgent craving for better understanding the mechanisms underpining the pathogenesis of T2DM and how to manage T2DM efficiently, particularly through drug administration. As a multifactoral disease, T2DM is driven by genetic, epigenetic, and nongenetic mechanisms, among which a substantial fraction are interdependent signaling pathways. A highly possible paradigm for various signaling pathways acting in T2DM, is where they serve as both the causes and consequences of T2DM progression and, function in an interacted manner rather than separately. In particular, environmental factors, together with genetic risks correlated to T2DM, are prone to elicit vibrations of the signaling pathways in the peripheral tissues and pancreatic islets. Interfering with these signaling pathways seems to engender outcomes that are conducive or obstructive to the pathological pertubances of T2DM, especially IR and β cell dysfunction. In terms of IR, modifications in these signaling pathways are likely to rely on their multifaceted interplays with the insulin/AKT to fluctuate the peripheral susceptibility to insulin action, despite the discrepancies in the specific roles of individual pathways. In terms of β cell dysfunction, signaling pathways also converge to regulating insulin secretion and fate of β cells, so intrinsic or acquired traits of these signaling pathways appear to partially dictate the adaptive capacity of β cells in response to metabolic demands. Importantly, many medications that normalize blood glucose levels and prevent or inverse diabetic complications have been identified to establish their pharmaceutical benefits via the mechanisms involving signaling pathways. One prominent example is the extensively used clinical antidiabetic drug, metformin, which acts through the AMPK pathway to powerfully improve metabolic disoders.^15^ Nevertheless, a fraction of drugs or compounds targeting signaling pathways have unexpected side‐effects or inadequate effectiveness, owing to additional pathophysiological roles or minor metabolic effects of signaling pathways. For example, TGFβ‐specific therapies have been witnessed to exert inevitable effects on immune system, thus slowering the progress in employing them for treating DN. For these reasons, discerning the functions and mechanisms of signaling pathways in T2DM would help ease the road to well‐managing T2DM with drugs.

However, there are several obstacles in studying signaling pathways and interventions of T2DM. Firstly, an unfortunate trend is excessively highlighting the pathological significance of a sole molecule in signaling pathways, which is intensified by the dramatic progress in genetic manipulation technologies. This misfortune of trend in large part relates to concerns that manipulating genes could inevitably cause adaptive or compensatory effects concealing the real effects of certain molecules or pathways in T2DM progression. Luckily, considerable progresses in omics methods, such as, spatially resolved genomics, transcriptomics, and metabonomics, make it available to rectify this trend. In particular, devising ways to assess genomic, proteomic, and metabolic status in prediabetic and diabetic tissues while preserving spatial information and single cell resolution, is of great propensity to delineate the inclusive but accurate molecular mechanisms of T2DM. Furthermore, biomolecular condensates or droplets have been increasingly identified as an interaction basis for the molecules in signaling pathways,^670^ which is less emphasized in the past studies of T2DM. Hence, it might be urgent to highlight the roles of liquid condensates or liquid–liquid phase separation in T2DM, which is possibly linked to abnormal protein aggregation. In addition, another important question to purse for the accurate roles of signaling pathways in T2DM, is to create in vitro models that sincerely mimic the real progression of T2DM. Nevertheless, feasible approaches to address this problem might be involved in the organoid technologies, which may largely recapitulate essential features of in vivo organ development and biological function. As such, they offer tractable and faithful in vitro tools for disentangling molecular mechanisms of T2DM and developing regenerative pancreatic islets, as well as confer a promising strategy that compensates for pharmacological therapies in advanced T2DM. Importantly, we anticipate that systematic application of these novel methodologies and notions in basic research and clinical translation of signaling pathways in T2DM would have a promising impact in contributing to the discovery of antidiabetic drugs with enhanced effectiveness and safety. This is possibly because precise interventions usually derive from precise investigations.

In summary, remarkable insights over the past few decades have gained vital understandings of signaling pathways related to T2DM and therapeutic interventions. Efficient management of T2DM and its complications is still challenging, but these understandings available now, together with more discoveries in the future, hold the potential to achieve more potent and specific drug interventions for T2DM and its complications. Hence, we hope that the knowledges summarized here can provide different ideas for researchers working in the field of T2DM, ultimately helping identify new therapeutic targets that could break the vicious development of this disease.

## AUTHOR CONTRIBUTIONS

Y. T. and X. F. conceived the idea; R. C., H. T., Y. Z., G. L., H. X., and G. R. performed the literature search and drafted the manuscript; R. C., H. T., and Y. Z. revised and edited the manuscript; X. F. and Y. T. supervised and revised the manuscript. All the authors have read and approved the final manuscript.

## CONFLICT OF INTEREST STATEMENT

All the authors declared no conflict of interest.

## ETHICS STATEMENT

Not applicable.

## Data Availability

Not applicable.
